# Learning generalizable AI models for multi-center histopathology image classification

**DOI:** 10.1038/s41698-024-00652-4

**Published:** 2024-07-19

**Authors:** Maryam Asadi-Aghbolaghi, Amirali Darbandsari, Allen Zhang, Alberto Contreras-Sanz, Jeffrey Boschman, Pouya Ahmadvand, Martin Köbel, David Farnell, David G. Huntsman, Andrew Churg, Peter C. Black, Gang Wang, C. Blake Gilks, Hossein Farahani, Ali Bashashati

**Affiliations:** 1 School of Biomedical Engineering, University of British Columbia, Vancouver, BC Canada; 2 Department of Electrical and Computer Engineering, University of British Columbia, Vancouver, BC Canada; 3 Department of Pathology and Laboratory Medicine, University of British Columbia, Vancouver, BC Canada; 4 Vancouver General Hospital, Vancouver, BC Canada; 5 Department of Urologic Sciences, University of British Columbia, Vancouver, BC Canada; 6 Department of Pathology and Laboratory Medicine, University of Calgary, Calgary, AB Canada; 7 BC Cancer Research Institute, Vancouver, BC Canada

**Keywords:** Cancer imaging, Diagnostic markers

## Abstract

Investigation of histopathology slides by pathologists is an indispensable component of the routine diagnosis of cancer. Artificial intelligence (AI) has the potential to enhance diagnostic accuracy, improve efficiency, and patient outcomes in clinical pathology. However, variations in tissue preparation, staining protocols, and histopathology slide digitization could result in over-fitting of deep learning models when trained on the data from only one center, thereby underscoring the necessity to generalize deep learning networks for multi-center use. Several techniques, including the use of grayscale images, color normalization techniques, and Adversarial Domain Adaptation (ADA) have been suggested to generalize deep learning algorithms, but there are limitations to their effectiveness and discriminability. Convolutional Neural Networks (CNNs) exhibit higher sensitivity to variations in the amplitude spectrum, whereas humans predominantly rely on phase-related components for object recognition. As such, we propose Adversarial fourIer-based Domain Adaptation (AIDA) which applies the advantages of a Fourier transform in adversarial domain adaptation. We conducted a comprehensive examination of subtype classification tasks in four cancers, incorporating cases from multiple medical centers. Specifically, the datasets included multi-center data for 1113 ovarian cancer cases, 247 pleural cancer cases, 422 bladder cancer cases, and 482 breast cancer cases. Our proposed approach significantly improved performance, achieving superior classification results in the target domain, surpassing the baseline, color augmentation and normalization techniques, and ADA. Furthermore, extensive pathologist reviews suggested that our proposed approach, AIDA, successfully identifies known histotype-specific features. This superior performance highlights AIDA’s potential in addressing generalization challenges in deep learning models for multi-center histopathology datasets.

## Introduction

Visual microscopic study of diseased tissue by pathologists has been the cornerstone in cancer diagnosis and prognostication for more than a century. Hematoxylin and eosin (H*&*E)-staining is the most common and standard method for examining tissues under microscope, in which Hematoxylin dyes the cell nuclei a dark purple color, and the Eosin produces a pink color on the other structures including the extracellular matrix and cytoplasm^1–3^. Digital pathology is gaining prominence with the extensive adoption of specialized scanning devices, producing Whole Slide Images (WSIs) by digitizing specimens, thereby making them well-suited to sophisticated deep learning algorithms, a subset of AI, for automated decision support.

The use of deep learning algorithms has shown remarkable potential in the assessment of digitized histopathology slides for diagnostics and biomarker discovery, enabling earlier and more precise diagnoses and treatments. By leveraging deep learning algorithms, researchers have achieved accurate classification, grading, and outcome prediction of diverse cancer types, such as prostate^4^, gastric^5^, lung^6^, breast^7^, endometrial^8^, bladder^9^ and colorectal^10^ cancers, based on histopathology images. As an example, a deep learning-based model^4,11^ has demonstrated the ability to perform Gleason grading of prostatic adenocarcinomas with high accuracy, comparable to that of pathologists, thus enabling accurate prognostic stratification of patients. Nevertheless, despite the achievements of these techniques, there exist challenges associated with their implementation and deployment in clinical settings.

Deep learning models tend to be data-intensive and require a significant amount of training data. The acquisition of a sufficient quantity of data from a single source is generally challenging, particularly for data like histopathology scans, due to various limitations including technical, ethical, and financial constraints as well as confidentiality concerns. In an ideal scenario, a network should be trained using data acquired from a single center, and subsequently applied to multiple centers. However, this can be challenging in histopathology sections due to inconsistent color appearances, known as domain shift. These inconsistencies arise from variations between slide scanners and different tissue processing and staining protocols across various pathology labs. While pathologists can adapt to such inconsistencies, deep learning-based diagnostic models often struggle to provide satisfactory results as they tend to overfit to a particular data domain^12–16^. In the presence of domain shift, domain adaptation is the task of learning a discriminative predictor by constructing a mapping between the source and target domains.

One approach to tackle this problem is labeling new images in the target domain and fine-tuning the trained model on source domain^17,18^, but this is time-consuming and costly, especially in biomedical fields where expert annotation is required. Another possible solution is to convert color images to grayscale^19,20^. However, such approaches exclude informative elements within the color space of images that might contribute to an accurate diagnosis.

Various techniques aim to mitigate generalization errors in histopathology images by manipulating color spaces, categorized into stain color augmentation and normalization. Augmentation simulates diverse stain variations for stain-invariant models, while normalization aligns training and test color distributions to reduce stain variation. Within the domain of color augmentation, methodologies range from basic techniques to advanced H*&*E-based approaches^21–23^. Typically, these methods involve direct modifications to images within the H*&*E color space, aiming to replicate specific variations in H*&*E staining. The color normalization techniques^12,24,25^ have received significant attention within the field of histopathology image analysis. The conventional methods within this domain aim to normalize the color space by estimating a color deconvolution matrix for identifying underlying stains^24,26^. Alternative advancements in stain style transfer encompass techniques like histogram matching^27,28^, CycleGAN^29–31^, style transfer^23^, and Network-based^22^. Notably, Tellez et al.^22^ introduced an image-to-image translation network that reconstructs original images from heavily augmented H*&*E images, facilitating effective stain color normalization in unseen datasets. In the most recent approaches self-supervised learning strategies^32,33^ have been proposed for color normalization.

While color normalization methods have been shown to improve the performance of target datasets, they suffer from two main drawbacks. Firstly, most color normalization approaches require the manual selection of a reference image; and this choice can substantially affect the performance of the models^12^. Secondly, deep learning models have been shown to recognize the tissue submission site even after deploying color normalization techniques. This was shown in a study by Howard et al.^34^, where they analyzed the differences in slide image characteristics from different centers using classical descriptive statistics. Their study revealed that all these statistics exhibited variance according to the tissue submitting center while color normalization methods could improve only some of these statistical characteristics and had no effect on the remainder. This suggests that these techniques do not necessarily remove all the site-specific signatures and therefore, may not lead to more generalizable models.

Adversarial networks^15^ have become prevalent in addressing the challenge of domain adaptation. By leveraging adversarial training techniques, domain adaptation models aim to bolster the model’s adaptability to diverse data distributions encountered during deployment. This is achieved through the alignment of feature distributions between the source and target domains. Central to adversarial domain adaptation is the feature extractor network, which operates with the dual objective of learning representations that are both discriminative for the primary task and invariant to domain shifts. Several studies have investigated the effectiveness of adversarial networks for domain adaptation in the realm of histopathology images^13,35,36^. In the works by Lafarge et al.^35^ and Otalora et al.^36^, adversarial networks are employed for domain adaptation tasks concerning mitosis detection in breast cancer histopathology images and Gleason pattern classification in prostate cancer. These approaches involve incorporating the adversarial network with color augmentation and stain normalization. However, it’s important to note that this integration may not be optimal, as the adversarial network should ideally demonstrate robust performance without relying on color normalization support. Ren et al.^13^ employed an adversarial network for the classification of low and high Gleason grades. A Siamese architecture was implemented as a regularization technique for the target domain. While this regularization demonstrated enhanced performance in the target domain, it necessitated the use of a distinct classifier for the source domain, rather than utilizing a shared feature representation network. Additionally, it is noteworthy that the integration of a Siamese architecture contributes to an increase in the computational time of the network.

Despite their potential, adversarial networks have certain limitations when applied to real-world applications^37–39^. First, a concern emerges regarding the potential hindrance of feature discriminability which results in lower performance when compared to supervised networks on target data^40^. Furthermore, these networks have not fully exploited transferability and concentrate only on distribution matching in the feature space by minimizing the statistical distance between domains while ignoring the class space alignment. As a result, the classifier may misclassify target samples that are close to the decision boundary or far from their class centers. Recently, self-supervised auxiliary tasks have been utilized to improve the performance of these networks in the context of histopathology images^13,32,41,42^. Although the self-supervised auxiliary tasks allow for the acquisition of more specific features pertaining to the color space of target data samples, these approaches are still grappling with the aforementioned issues regarding discriminability and transferability.

From a frequency domain perspective, it has been proven that CNNs benefit from the high-frequency components of the image, rendering them more sensitive to amplitude spectrum variations while humans, unlike CNNs, rely more on phase-related components for object recognition^43,44^. The color variations of histopathology images (i.e., domain shift) notably affect the amplitude spectrum of images, while phase-related components carry more informative content. In light of this, we hypothesize that incorporating frequency information has the potential to enhance both the discriminability and knowledge transferability of the domain adversarial networks. Therefore, we propose the Adversarial fourIer-based Domain Adaptation (AIDA) framework by integrating a module, referred to as FFT-Enhancer, into the feature extractor for patches (i.e., tiny portions of the whole image) to make the adversarial network less sensitive to changes in amplitude (i.e., variations in color space) and to increase the attention paid to phase information (i.e., shape-based features). The FFT-Enhancer module, with its straightforward calculation and minimal computational burden, emerges as an ideal candidate for this purpose. Furthermore, we specifically investigate the application of various convolutional layers for the purpose of domain adversarial training in histopathology images, revealing that features extracted from intermediate layers exhibit greater suitability for effective domain adaptation.

We conducted a thorough and all-encompassing investigation into subtype classification of histopathology datasets of ovarian, pleural, bladder, and breast cancers which encompass 1113, 247, 422, and 482 slides from various hospitals, respectively. The experimental findings conclusively demonstrate the superiority of AIDA over the baseline, color augmentation and normalization techniques, the conventional adversarial domain adaptation (ADA) network, and a pre-trained self-supervised model on a massive dataset. Additionally, our investigation reveals AIDA’s efficacy in discriminating histotypes within the feature space and shows its capacity for accurately identifying tumor regions and subtype-specific morphometric characteristics as assessed by expert pathologists. The demonstrated superiority of AIDA’s performance reaffirms its potential advantages in addressing challenges related to generalization in deep learning models when dealing with multi-center histopathology datasets.

## Results

### Proposed Adversarial fourIer-based Domain Adaptation (AIDA) framework

**Fig. 1:**
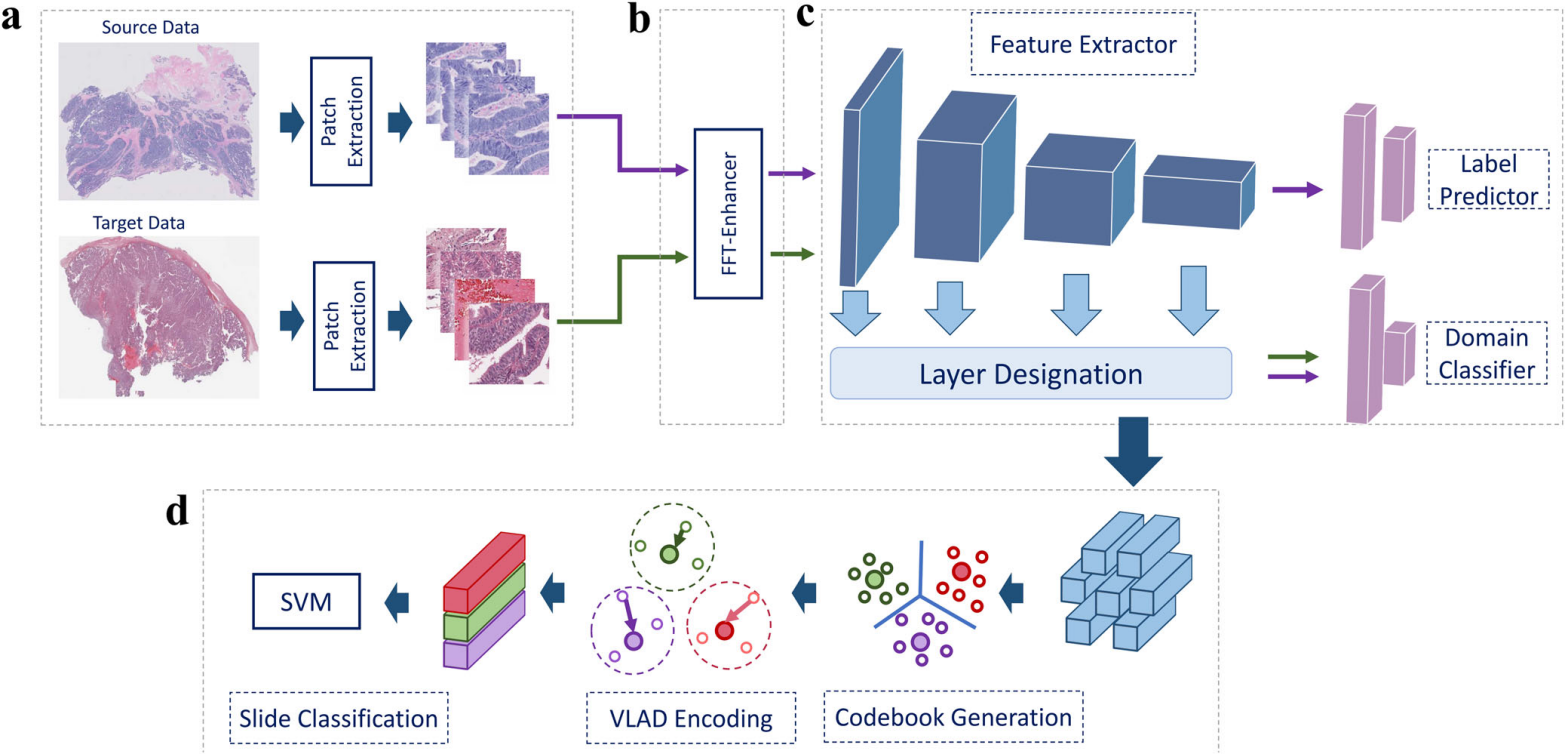
Overview of the proposed method (AIDA). **a** The patches are first extracted from the WSIs in both the source and target domains. **b** Through FFT-Enhancer, patch colors from the source domain are adjusted to look more like patches from the target domain. **c** The label predictor is trained using features derived from the source domain, whereas the domain classifier is optimized using features derived from both the source and target domains. **d** In order to predict slide-level labels, the extracted features are fed into the VLAD aggregation method.

The AIDA approach proposed in this study addresses the challenges associated with the classification of large WSIs originating from different centers, which exhibit domain shift, by combining adversarial training and the FFT-Enhancer module. As illustrated in Fig. 1, the AIDA approach consists of four key components. First, the WSIs are partitioned into small patches (Fig. 1a), which serve as input data for the network. The adversarial training component (Fig. 1c) comprises a feature extraction module, a label predictor, and a domain classifier. This component is designed to leverage input samples from both the source and target domains. Specifically, the label predictor is trained on source domain samples, while the domain classifier is trained on both source and target domain samples. To facilitate knowledge transfer from the target domain to the label predictor, we utilize the FFT-Enhancer module (Fig. 1b) which leverages the color space of the target domain.

The patch-level results obtained from the three aforementioned steps are then aggregated to produce slide-level results. We employ the Vector of Locally Aggregated Descriptors (VLAD)^45^ approach to generate slide-level features from extracted features of the patches within a slide. This method was selected due to its capability to efficiently aggregate local features into a condensed representation, thereby decreasing computational complexity while retaining discriminative information. VLAD encoding has proven successful in a variety of computer vision tasks, rendering it a fitting choice for our feature aggregation step. Finally, we apply the Support Vector Machine (SVM) classifier to classify the slides (Fig. 1d). Further details regarding the proposed network are provided in the Methods section.

Throughout the remainder of this paper, we compare a total of eight approaches: (a) baseline model (“Base”) where we employed a standard CNN, (b) Hematoxylin-Eosin-DAB color augmentation (“HED”)^22^, (c) Macenko color normalization (“Macenko”)^26^, (d) composite color normalization (“CNorm”)^12^, (e) a foundation model (“CTransPath”)^46^ as a self-supervised feature extractor, (f) a variant of the baseline approach, enriched with the FFT-Enhancer module (“Base-FFT”), (g) conventional adversarial domain adaptation network (“ADA”)^15^, and (h) our proposed method (AIDA). To ensure a fair comparison, we maintained identical backbone architectures (i.e., ResNet18) for all the aforementioned methods.

AIDA’s performance was evaluated across four datasets: ovarian, pleural, bladder, and breast cancers. The ovarian cancer dataset contains 1053 Whole Slide Images (WSIs) from 523 patients in the source domain and 60 WSIs from 60 patients in the target domain, encompassing five primary histotypes. The pleural dataset comprises 194 WSIs from 128 patients in the source domain and 53 WSIs from 53 patients in the target domain, covering both benign and malignant cases from two centers. For the bladder dataset, 262 WSIs from 86 patients were used in the source domain, while 160 WSIs from 72 patients were used in the target domain, including two distinct histological subtypes. In the breast cancer dataset, the public ICIAR-2018 dataset^47^ was utilized for the source domain, containing 400 breast biopsy images from 257 patients. For the target domain, the public BreaKHis dataset^48^, consisting of 7909 small biopsy slides of breast tissue, was employed. The breast dataset was analyzed for two subtypes: benign and malignant. Notably, the source and target domains for all datasets originate from distinct hospitals. For further details on these datasets, refer to the Data section and Table 1.

**Table 1:** Overview of datasets

Dataset	Domain	*#*Slides	*#*Patients	Patch size	Magnification	Classes		
						Subtype	*#*Patients	*#*Slides
Ovarian Cancer	Source	1053	522	512 × 512	20X	CCOC	97	173
						ENOC	119	257
						HGSC	212	446
						LGSC	36	76
						MUC	59	101
	Target	60	60	512 × 512	20X	CC	10	10
						ENOC	10	10
						HGSC	31	31
						LGSC	4	4
						MUC	5	5
Pleural Cancer	Source	194	130	512 × 512	40X	Benign	73	111
						Malignant	57	83
	Target	53	42	512 × 512	40X	Benign	17	27
						Malignant	25	26
Bladder Cancer	Source	262	86	512 × 512	20X	UCC	57	134
						MPC	29	128
	Target	160	72	512 × 512	20X	UCC	48	72
						MPC	24	88
Breast Cancer	Source	400	257	230 × 230	20X	Benign	129	200
						Malignant	131	200
	Target	82	82	230 × 230	20X	Benign	24	24
						Malignant	58	58

Across all four datasets, AIDA exhibited superior performance in the target domain, attaining balanced accuracy scores of 75.82%, 82.56%, 77.65%, and 74.11% for the ovarian, pleural, bladder, and breast cancer datasets, respectively. Further experimental details are provided in the subsequent sections.

### Designating the optimal CNN layer for the domain discriminator in AIDA

It is common practice to extract features from the final convolutional layer, although using earlier layers as the feature extractor is possible. In convolutional networks, the initial layers are responsible for detecting low-level features. Their small local receptive fields limit the context they can encode, resulting in the learning of only basic and minor details of images. As a result, these layers lack sufficient information for the domain discriminator and cannot capture the high-level semantic information needed to differentiate between domains. By contrast, the last convolutional layer builds up high-level features on top of low-level ones to detect larger structures in images. As a result, these features rely heavily on semantic and classification information from labeled data, i.e., the source domain. Nonetheless, they may have already learned a biased representation unsuitable for the target domain, thereby presenting a risk that these layers may not be able to learn from the target data. Accordingly, depending on the layers and features used, domain classifiers may encounter difficulties in constructing a feature space resilient to different domains.

**Fig. 2:**
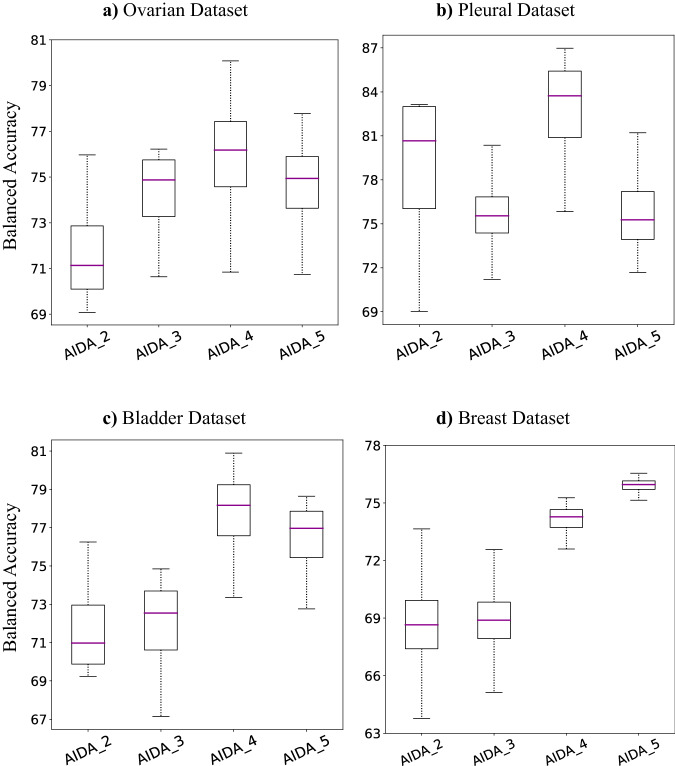
AIDA with different discriminator inputs. Comparison of the balanced accuracy achieved by using different layers as the input to the discriminator for the target domain of (**a**) the Ovarian Dataset, **b** the Pleural Dataset, **c** the Bladder Dataset, and (**d**) the Breast Dataset. In box plots, the central line represents the median, while the bottom and top edges of the box correspond to the 25^th^ and 75^th^ percentiles, respectively. (AIDA-X indicates using the *X*^th^ block as the input to the discriminator).

In order to assess the efficacy and utility of different layers as feature extractors, we constructed a domain classifier exploiting the output of the *X*^*th*^ convolutional block. We refer to these classifiers as AIDA-X, where *X* ∈ [2, . . . , 5]. The experimental results are presented in Fig. 2 for the Ovarian, Pleural, Bladder, and Breast datasets. In the initial three datasets, AIDA-4 exhibited superior performance in target-domain classification, except for the Breast dataset, where AIDA-5 outperformed it. However, the performance gap for the Breast dataset was minimal, with an estimated difference of approximately 1%, indicating that both AIDA-4 and AIDA-5 exhibited comparable performance on this dataset. This suggests that the fourth convolutional block contributes to more generalizable and optimal features for the domain classifier. To simplify our discussion, we will use the shorthand “AIDA” instead of “AIDA-4” throughout the paper, including when referring to the Breast dataset.

### The utilization of adversarial neural network, color augmentation, and color normalization exceeds the performance of the baseline

Various color normalization and augmentation techniques have been developed to address the challenge of color variation. In a recent study^12^, the effectiveness of different color normalization approaches was evaluated in the context of histopathology image classification. Their research revealed that employing a combination of color normalization methods with multiple reference images yielded the most consistent results. Therefore, we adopted this approach, which involves integrating Reinhard^24^, Macenko^26^, and Vahadane^49^ methods with several reference images. This combined approach, referred to as CNorm throughout the paper, was utilized to assess the performance of AIDA against other color normalization techniques.

In another study Tellez et al.^22^ compared various color normalization and augmentation approaches for classifying histopathology images with color variations. Among these approaches, the HED color augmentation method was found to outperform other color normalization and augmentation approaches across several datasets. Consequently, we integrated this method into our comparison framework, referring to it as HED for clarity and consistency. Additionally, we employed the Macenko method as a standalone color normalization approach using only one reference image.

In AIDA, we employed adversarial training in conjunction with the FFT-Enhancer module. In this section, we present the results obtained from the adversarial training component, specifically ADA, and compare its performance with other approaches. This allows us to assess the individual contributions of adversarial training and the FFT-Enhancer module to the overall performance of AIDA. The ADA method employed in our study is based on the concept of adversarial domain adaptation neural network^15^. To ensure a fair comparison with AIDA, we followed the approach of using the output of the fourth layer of the feature extractor to train the domain discriminator within the network.

Given that all datasets are imbalanced with respect to the distribution of cancer histotypes, we predominantly utilized the slide-level balanced accuracy metric to compare the performance of various methods in the rest of this paper. In our study, we applied HED, Macenko, CNorm, and ADA to all datasets. Our findings (Supplementary Table 1) reveal that in the target domain of the Ovarian dataset, all HED, Macenko, CNorm, and ADA outperformed the Base method with a balanced accuracy of 67.07%, 73.49%, 72.14%, and 74.16%, respectively, as compared to Base’s balanced accuracy of 64.65%. Notably, Macenko, CNorm, and ADA demonstrated similar performance levels, while HED exhibited a notably lower accuracy. Conversely, in the source domain of the Ovarian dataset, all methods showed comparable performance. For the target domain of the Pleural dataset (Supplementary Table 2), Macenko (80.96%), CNorm (79.55%), and ADA (79.72%) outperformed the Base method (76.70%), while HED (76.80%) showed similar performance to the Base. In the source domain of the Pleural dataset, HED, Macenko, and ADA achieved nearly identical balanced accuracy, all surpassing CNorm’s performance.

In the case of the Bladder dataset (Supplementary Table 3), the HED, Macenko, CNorm, and ADA approaches exhibited superior performance compared to the Base approach in the target domain. They achieved balanced accuracies of 57.66%, 66.42%, 73.73%, and 73.15%, respectively, while the Base approach obtained a performance of 54.77%. Notably, ADA and CNorm outperformed Macenko and HED in this dataset, with HED showing marginal improvement over the Base. In the source dataset, HED, Macenko, and CNorm yielded similar results, slightly outperforming ADA. In the Breast dataset (Supplementary Table 4), all methods - HED, Macenko, CNorm, and ADA - surpassed the Base performance of the target dataset. They achieved balanced accuracies of 57.57%, 58.91%, 65.06%, and 60.49%, respectively, compared to the Base’s performance of 55.15%. Notably, CNorm stood out as the most effective approach among the four methods. ADA and Macenko demonstrated similar performance levels, while HED showed marginal improvement over the Base, akin to the Pleural dataset. In the source domain, HED, CNorm, and ADA outperformed the Base performance, while Macenko closely matched the Base’s performance. Additionally, to conduct a statistical comparison of these methods, we computed the p-values using the Wilcoxon signed-rank method (two-sided) and visualized them in Supplementary Fig. 1.

Overall, across all datasets, Macenko, CNorm, and ADA consistently improved the performance of the target datasets to a greater extent than HED, with a notable margin. Furthermore, all methods demonstrated minimal impact on the performance of the source domain.

### Incorporating the FFT-Enhancer in the networks boosts their performance

**Fig. 3:**
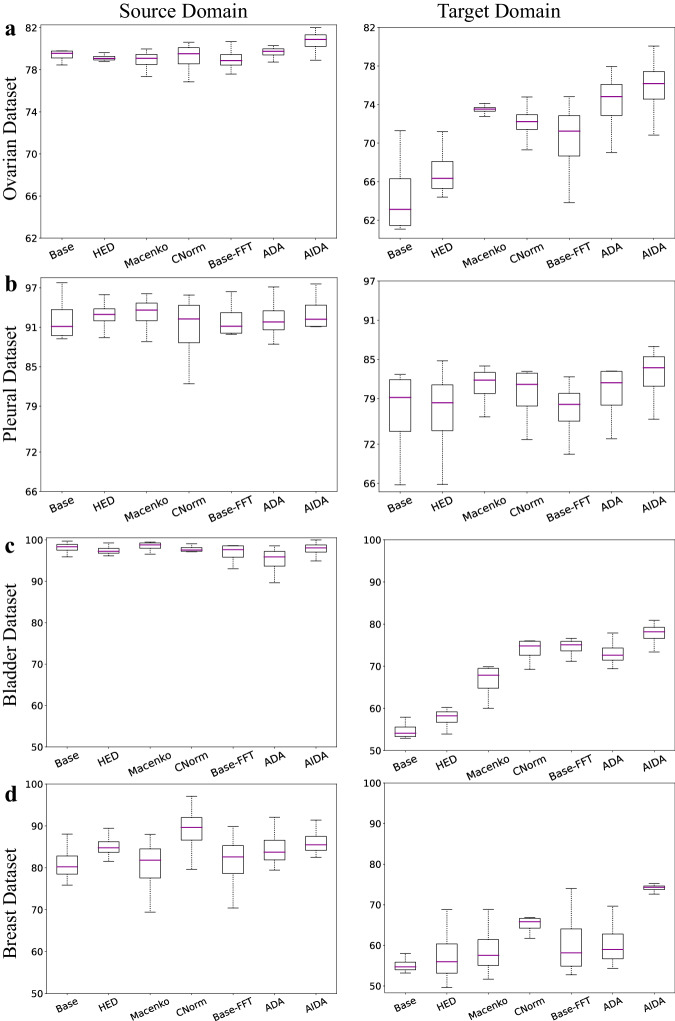
Comparative analysis: AIDA versus other methods. The balanced accuracy comparison of the proposed AIDA model with the Base, HED, Macenko, CNorm, Base-FFT, and ADA for the source (left column) and target (right column) domains of (**a**) the Ovarian dataset, **b** the Pleural dataset, **c** the Bladder dataset, and (**d**) the Breast dataset. In box plots, the central line represents the median, while the bottom and top edges of the box correspond to the 25^th^ and 75^th^ percentiles, respectively.

Figure 3a illustrates the distribution of the balanced accuracy for seven approaches of: Base, HED, Macenko, CNorm, Base-FFT, ADA, and AIDA, on the source and target domains of the Ovarian cancer dataset, respectively. The utilization of the FFT-Enhancer (Base-FFT) improved the balanced accuracy of the Base on the target domain of the Ovarian dataset from 64.65% to 70.28% (*p*-value = 6.71e−3). According to the experimental results presented in Supplementary Table 1, it can be inferred that AIDA achieved superior performance across various metrics, such as balanced accuracy, Cohen’s Kappa, F1-score, and Area Under the ROC Curve (AUC), for both the source and target domains. Specifically, AIDA yielded the highest balanced accuracy (75.82%), surpassing the performance of Base (*p*-value = 6.0e−5), HED (*p*-value = 3.1e−4), Macenko (*p*-value = 1.51e−1), CNorm (*p*-value = 3.53e−2), Base-FFT (*p*-value = 2.01e−3), and ADA (*p*-value = 2.80e−2). The results of the study demonstrate that AIDA not only outperformed the other approaches in the target domain but also in the source data with a balanced accuracy of 80.68%. This suggests that the inclusion of the FFT-Enhancer module has enhanced the label predictor’s ability to adapt to the source domain.

Figure 3b presents a comparison of the balanced accuracy of seven approaches applied to the Pleural dataset. Consistent with the findings on the Ovarian dataset, our proposed AIDA approach demonstrated superior performance in terms of various metrics for both the source and target domains. Specifically, AIDA demonstrated the best performance among all methods, achieving a balanced accuracy of 82.56% compared to Base (*p*-value = 9.16e−3), CNorm (*p*-value = 4.79e−2), HED (*p*-value = 3.36e−3), Macenko (*p*-value = 4.54e−1), Base-FFT (*p*-value = 5.37e−3), and ADA (*p*-value = 4.13e−2). In addition, the incorporation of the FFT-Enhancer module resulted in improved performance for the Base method, with Base-FFT achieving a balanced accuracy of 77.24%. A similar outcome was achieved in the source domain as well, with 93.28% balanced accuracy associated with AIDA. It is noteworthy that, among all approaches applied to the Pleural dataset, AIDA was the only method that outperformed the Base method.

In Fig. 3c, the distributions of the balanced accuracy for seven different approaches on the Bladder dataset are presented for the source and target domains. Similar to the other two datasets, the AIDA method exhibited the most favorable results on the target domain, with a balanced accuracy of 77.65%, outperforming the Base (*p*-value = 6.0e−5), HED (*p*-value = 1.2e−4), Macenko (*p*-value = 4.27e−3), CNorm (*p*-value = 8.36e−3), Base-FFT (*p*-value = 6.71e−3), and ADA (*p*-value = 2.15e−2) approaches. Furthermore, incorporating the FFT-Enhancer technique in the Base-FFT approach led to improved performance, achieving a balanced accuracy of 74.48% compared to the Base’s performance of 54.77%. It is important to note that while all methods showed improved performance on the target domain, they were unable to reach the same level of performance as the Base on the source domain, with the exception of Macenko. This can be attributed to the Base network’s excessive overfitting on the source domain, which consequently led it to function as a random classifier on the target domain. However, it’s worth mentioning that there exists a slight disparity between the performance of the Base model on the source domain and that of the other methods.

Figure 3d illustrates a comparative analysis of the balanced accuracy of seven approaches applied to the Breast dataset. Mirroring the observations on other datasets, our proposed AIDA approach exhibited superior performance across various metrics in the target domain. Specifically, AIDA outperformed all other methods, achieving a balanced accuracy of 74.11% compared to Base (*p*-value = 6.0e−5), HED (*p*-value = 1.2e−4), Macenko (*p*-value = 6.0e−5), CNorm (*p*-value = 8.5e−4), Base-FFT (*p*-value = 1.16e−3), and ADA (*p*-value = 1.8e−4). Furthermore, the integration of the FFT-Enhancer module led to improved performance for the Base method, with Base-FFT achieving a balanced accuracy of 60.75%. Notably, AIDA also excelled in the source domain, achieving the second-highest performance after CNorm (88.98%) with a balanced accuracy of 86.21%.

### AIDA outperforms ADA across different backbone architectures

In addition to employing ResNet18 as the backbone architecture in our previous experiments, we sought to further assess the efficacy of AIDA by training it with two alternative backbone architectures: MobileNetV3^50^ and Vision Transformer (ViT)^51^. Supplementary Table 5 presents a comparison of the results obtained by AIDA and ADA across these different backbone architectures. Remarkably, irrespective of the backbone utilized, AIDA consistently surpassed ADA in terms of balanced accuracy within the target domain. In the source domain of the Ovarian, Pleural, and Breast datasets, AIDA surpassed ADA, whereas, for the Bladder dataset, their performance was nearly identical. This comprehensive experiment reaffirms the consistent and superior performance of AIDA over ADA across various backbone architectures.

### Exploring the role of foundation models in AIDA

In recent studies, researchers have introduced several foundational models designed as feature extraction modules for histopathology images^46,52–54^. Typically, these models undergo training on extensive datasets containing diverse histopathology images. To enhance the extraction of histopathology-oriented features and investigate the role of foundational models, we chose to integrate CTransPath^46^, a self-supervised learning (SSL) foundational model that has demonstrated notable efficacy across several cancer types, into our methodology.

CTransPath employs a semantically relevant contrastive learning (SRCL) framework and a hybrid CNN-Transformer backbone to address the limitations of traditional SSL approaches. The SRCL framework selects semantically matched positives in the latent space, providing more visual diversity and informative semantic representations than conventional data augmentation methods. The hybrid backbone combines CNNs to extract local features and Transformers to capture global dependencies, ensuring stability and improved performance in histopathological image analysis. This architecture leverages a vast dataset, including around 15 million cancer genome atlas (TCGA) patches and pathology AI platform (PAIP) datasets, making it a robust and universal feature extractor for our adversarial domain adaptation model.

Our experimental setup involved several approaches utilizing CTransPath. 1) Initially, we employed CTransPath to extract features from image patches, which were then aggregated using a VLAD encoder to produce slide-level results. 2) We integrated CTransPath as the backbone feature extractor in both ADA and AIDA frameworks. Using pre-trained weights from CTransPath, we trained the entire networks with the inclusion of data augmentation techniques during training. 3) Similarly to the second approach, we utilized CTransPath as the feature extraction backbone in ADA and AIDA, but without applying any data augmentation techniques. The first experiment aimed to evaluate the performance of a foundation model, trained in a self-supervised manner on a large set of histopathology samples, when applied to our dataset. The second and third experiments were designed to investigate two specific aspects: first, the impact of using domain-specific pre-trained weights, obtained from large histopathology datasets, as opposed to conventional ImageNet weights; and second, the effect of general augmentation methods, such as rotation, flipping, and color jittering, on the performance of the network.

The results of these experiments are presented in Supplementary Table 6. Despite its promising architecture, our evaluation of CTransPath’s impact on model performance yielded mixed outcomes. CTransPath achieved balanced accuracy scores of 49.41%, 69.13%, and 64.60% on the target domains of the Ovarian, Pleural, and Breast datasets, respectively, which were lower than the performance of AIDA on these datasets. These results underscore the importance of domain adaptation in addition to efforts through building domain agnostic representation models (e.g., foundational models).

However, for the Bladder dataset, CTransPath achieved a balanced accuracy of 79.87%, surpassing the performance of AIDA (63.42%). Using CTransPath as a feature extractor yields superior performance to AIDA, even when employing domain-specific pre-trained weights as the backbone. However, upon closer examination of the results, we observed that the performance of CTransPath for the micropapillary carcinoma (MPC) subtype is 87.42%, whereas this value rises to 95.09% for AIDA (using CTransPath as the backbone). In bladder cancer, patients with MPC subtypes are very rare (2.2%)^55^, despite this subtype being a highly aggressive form of urothelial carcinoma with poorer outcomes compared to the urothelial carcinoma (UCC) subtype. Thus, our primary concern is accurately identifying MPC cases, prioritizing a higher positive prediction rate. In this context, the positive predictive value of AIDA (95.09%) surpasses that of CTransPath (87.42%), aligning with our objective of achieving higher sensitivity in identifying MPC cases.

Moreover, it is important to note that MPC slides typically exhibit a UCC background with usually small regions of micropapillary tumor areas. In this study, we used these slides as training data without any pathologists’ annotations, leading to the extraction of both UCC and MPC patches under the MPC label. Consequently, when fine-tuning the model with our source data, the network incorrectly interprets UCC patches as belonging to the MPC class, resulting in a tendency to misclassify UCC samples as MPC. While the primary objective of this study was domain adaptation rather than enhancing performance on the bladder dataset, future work will address this challenge by incorporating tumor annotations from expert pathologists to improve the model’s accuracy in classifying UCC samples.

In the second and third experiments, we demonstrated that AIDA consistently outperformed ADA, even when utilizing CTransPath with domain-specific pre-trained weights as the feature extractor. Specifically, AIDA achieved balanced accuracy scores of 80.93%, 72.95%, 63.42%, and 75.23% for the Ovarian, Pleural, Bladder, and Breast datasets, respectively. This demonstrates AIDA’s superior robustness and effectiveness compared to ADA in enhancing feature extraction capabilities, irrespective of the network’s initial weights.

Using CTransPath instead of ResNet18 backbone boosts the performance of AIDA on the target domains of two datasets of Ovarian and Breast. Specifically, on the Ovarian dataset, AIDA with CTransPath achieved 80.93% which is 5% better than AIDA with ResNet backbone (75.82%). While for the Pleural and Bladder datasets, the ResNet18 backbone was more successful. Similar to AIDA, CTransPath helped ADA to work better for the Ovarian and Breast datasets while ADA with ResNet18 backbone resulted in better performance for the Pleural and Bladder datasets. CTransPath’s hybrid architecture, which combines local fine structure extraction with global contextual understanding, appears to be particularly well-suited for the Ovarian and Breast datasets. These datasets likely benefit from the domain-specif pre-trained weights and the model’s ability to capture nuanced morphological details and broader contextual information. On the other hand, the Pleural dataset might have features that are more effectively captured by ResNet18’s traditional convolutional approach.

In the third experiment involving CTransPath, we conducted training without employing regular augmentations. Across the Ovarian, Pleural, Bladder, and Breast datasets, AIDA without augmentation functions yielded classification accuracies of 82.67%, 73.77%, 64.56%, and 77.45% respectively, surpassing its augmented counterpart. Conversely, ADA with the CTransPath backbone exhibited superior performance when trained with augmentation. The distinguishing factor between AIDA and ADA lies in the inclusion of the FFT-Enhancer module. Our findings indicate that when utilizing a backbone with domain-specific pre-trained weights, the FFT-Enhancer can enhance model performance without augmentation, surpassing its augmented counterpart. This outcome may be attributed to CTransPath’s extensive training on a diverse array of histopathology images, enabling adaptation to various general variations, including those related to color. Consequently, the pre-trained weights enable the model to accommodate samples with distinct color spaces, with the FFT-Enhancer aiding in sharpening the focus on tumor morphology and shape during training.

### Cancer histotypes can be readily discerned within the feature space of AIDA

**Fig. 4:**
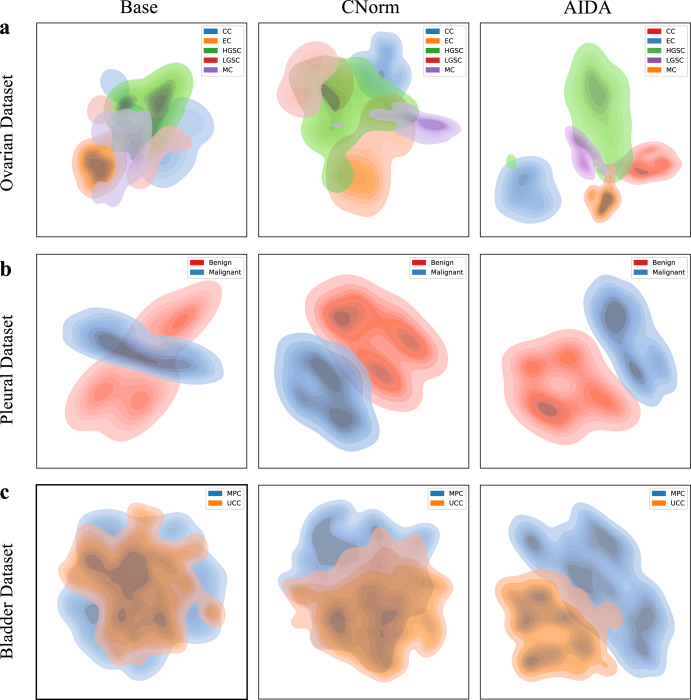
Comparing feature space representations using tSNE. Visualizing the feature spaces of Base, CNorm, and AIDA for datasets related to (**a**) Ovarian Cancer, **b** Pleural Cancer, and (**c**) Bladder Cancer.

The t-Distributed Stochastic Neighbor Embedding (t-SNE) approach was employed to visually represent the joint feature space of the source and target domains learned through the use of Base, CNorm, and AIDA. Figure 4 shows the t-SNE results, with the first, second, and third rows representing the Ovarian, Pleural, and Bladder datasets, respectively. According to the findings, CNorm outperformed Base, while AIDA is the most effective method in separating the five histotypes of ovarian cancer. Similarly, the representation of the feature space of the Pleural and Bladder datasets showed that AIDA outperformed the other approaches in generating more discriminative features for subtype classification.

### Effectiveness of AIDA through the visualization of the spatial distribution of tumor regions

Heatmaps were generated for each WSI to visualize the spatial distribution of tumors. This was accomplished by converting the prediction probability results of each patch into colors on WSI heatmaps. A higher classification score in tumor prediction is represented by a closer color to red in the heatmap image, indicating a higher likelihood of a tumor diagnosis.

**Fig. 5:**
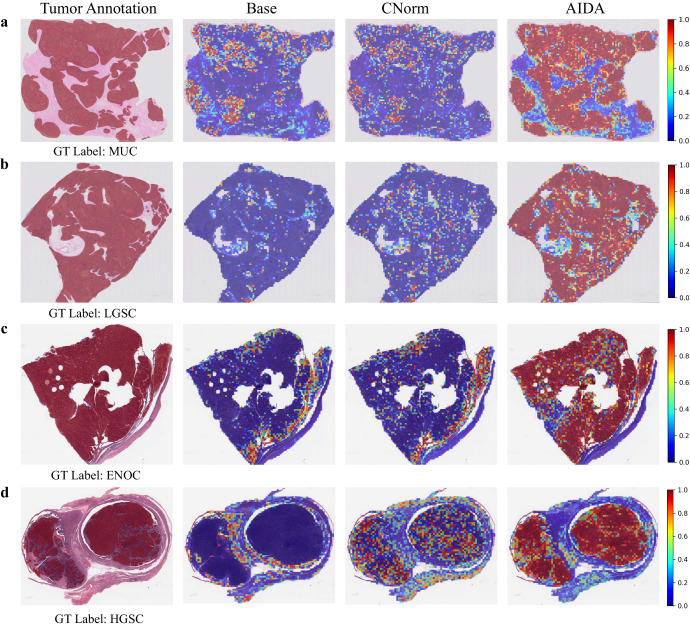
Comparative heatmap visualization of Ovarian dataset. Heatmap analysis of samples (**a**, **b**) from the source domain and (**c**, **d**) from the target domain of the Ovarian cancer dataset. The first column is the input slide incorporating the tumor annotation provided by the pathologist, and the second to fourth columns are the outputs of Base, CNorm, and AIDA methods. The closer it is to red, the more likely it is to be classified as a ground truth label, while the closer it is to blue, the less likely it is.

In Fig. 5, we compare the heatmaps generated by the proposed AIDA with those generated by the Base and CNorm for selected samples from both source (a and b) and target (c and d) domains of the Ovarian dataset. The results show that AIDA outperforms the other methods. For instance, Fig. 5a shows a sample that has been diagnosed as “MUC”. However, the Base and CNorm classified most of the patches as other subtypes, detecting only a few patches with “MUC”, leading to a misclassification of the entire slide as “ENOC”. In contrast, AIDA could accurately classify the majority of the patches as “MUC” with high probabilities, as evidenced by the high red intensities on the heatmap. Moreover, upon careful examination of Fig. 5, it becomes evident that the produced heatmaps by AIDA align precisely with the tumor annotations provided by the pathologist. This close correspondence serves as compelling evidence of AIDA’s proficiency in accurately visualizing the tumor area which underscores the capacity of AIDA to effectively capture and represent the tumor regions.

**Fig. 6:**
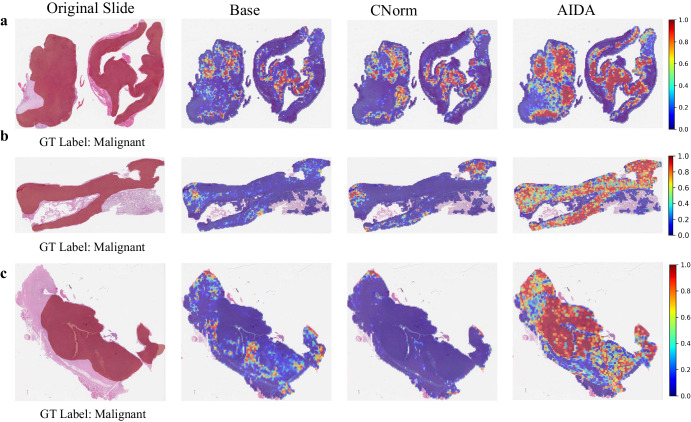
Comparative heatmap visualization of Pleural dataset. Heatmap analysis of samples (**a**) from the source domain and (**b, c**) from the target domain of the Pleural cancer dataset. The first column is the input slide incorporating the tumor annotation provided by the pathologist, and the second to fourth columns are the outputs of Base, CNorm, and AIDA methods. The closer it is to red, the more likely it is to be classified as a ground truth label, while the closer it is to blue, the less likely it is.

Figure 6 compares the heatmaps generated by the proposed AIDA, with those produced by the Base and CNorm for the source (a) and target (b and c) domains of the Pleural dataset. While all samples were diagnosed as “Malignant”, both the Base and CNorm approaches incorrectly classified the slides as “Benign”. However, by applying AIDA, the majority of patches were accurately classified as “Malignant”, ultimately leading to the correct classification of the entire slide as “Malignant”. Similar to the observations made in the context of ovarian cancer, a notable alignment is observed between the tumor annotations provided by the pathologist and the heatmap generated by the AIDA method for pleural cancer. This alignment demonstrates that our network possesses the capability to accurately localize tumor regions on the slide for pleural cancer.

**Fig. 7:**
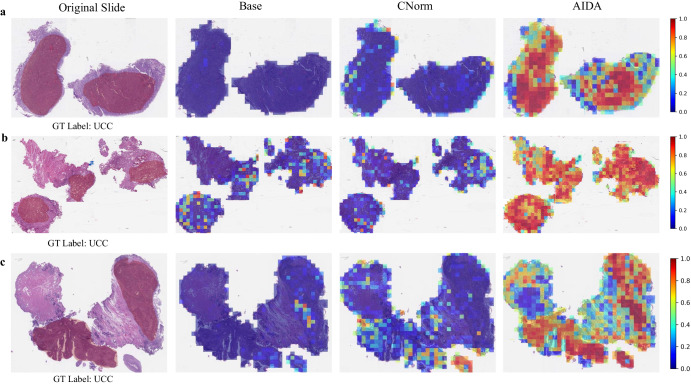
Comparative heatmap visualization of Bladder dataset. Heatmap analysis of three samples (**a**–**c**) from the target domain of the Bladder cancer dataset. The first column is the input slide incorporating the tumor annotation provided by the pathologist, and the second to fourth columns are the outputs of Base, CNorm, and AIDA methods. The closer it is to red, the more likely it is to be classified as a ground truth label, while the closer it is to blue, the less likely it is.

Figure 7 illustrates a comparison between the prediction heatmaps associated with the AIDA, Base, and CNorm methods for three samples (a–c) from the target domain. The results indicate that a significant number of patches within these slides were inaccurately identified as “MPC" by the Base and CNorm approaches, leading to the misclassification of these samples, which were in fact diagnosed as “UCC". Conversely, AIDA successfully classified the majority of patches in these slides as “UCC", which in turn enabled the model to correctly classify the entire slides. A thorough comparison between the annotations provided by the pathologist and the heatmaps generated by the proposed AIDA method indicated that AIDA demonstrates a commendable ability to proficiently localize tumor regions for bladder cancer, akin to its performance in ovarian and pleural cancers. This finding underscores the robustness and generalizability of AIDA in accurately identifying and delineating tumor areas It is noteworthy to highlight that in our analysis we specifically visualized the model output for the UCC subtypes. This deliberate focus stems from the observation that the other approaches employed in the study exhibited overfitting tendencies on the MPC subtypes, resulting in an accurate classification of all MPC slides. By emphasizing the visualization of the UCC subtypes, we aimed to explore the performance and generalizability of the models.

**Fig. 8:**
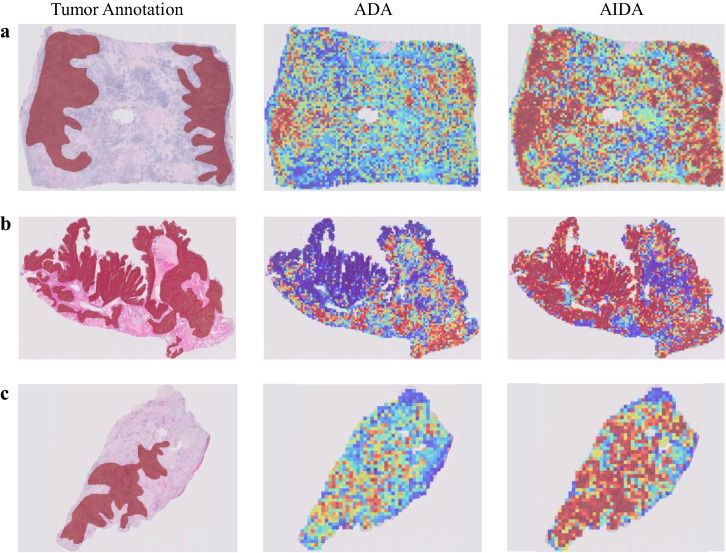
Comparative heatmap visualization of ADA and AIDA. Heatmap analysis (**a**–**c**) of three samples from the Ovarian dataset correctly classified by both ADA and AIDA methods. The first column is the input slide incorporating the tumor annotation provided by the pathologist, and the second and third columns are the outputs of ADA and AIDA methods. The closer it is to red, the more likely it is to be classified as a ground truth label, while the closer it is to blue, the less likely it is.

To gain deeper insights into the enhancement brought about by FFT-Enhancer on model performance, we examine the heatmaps generated by both ADA and AIDA for three samples that were accurately classified by both methods, as depicted in Fig. 8. While both approaches achieved the correct classification for these samples, a noticeable distinction arises in the heatmap output. Notably, the heatmap produced by AIDA demonstrates a closer resemblance to the annotated areas. This suggests that AIDA exhibits a higher proficiency in accurately classifying a majority of patches within the annotated regions compared to ADA.

### Identifying the most influential parts of an image captured by AIDA can lead to pinpointing subtype-specific morphometric features

**Fig. 9:**
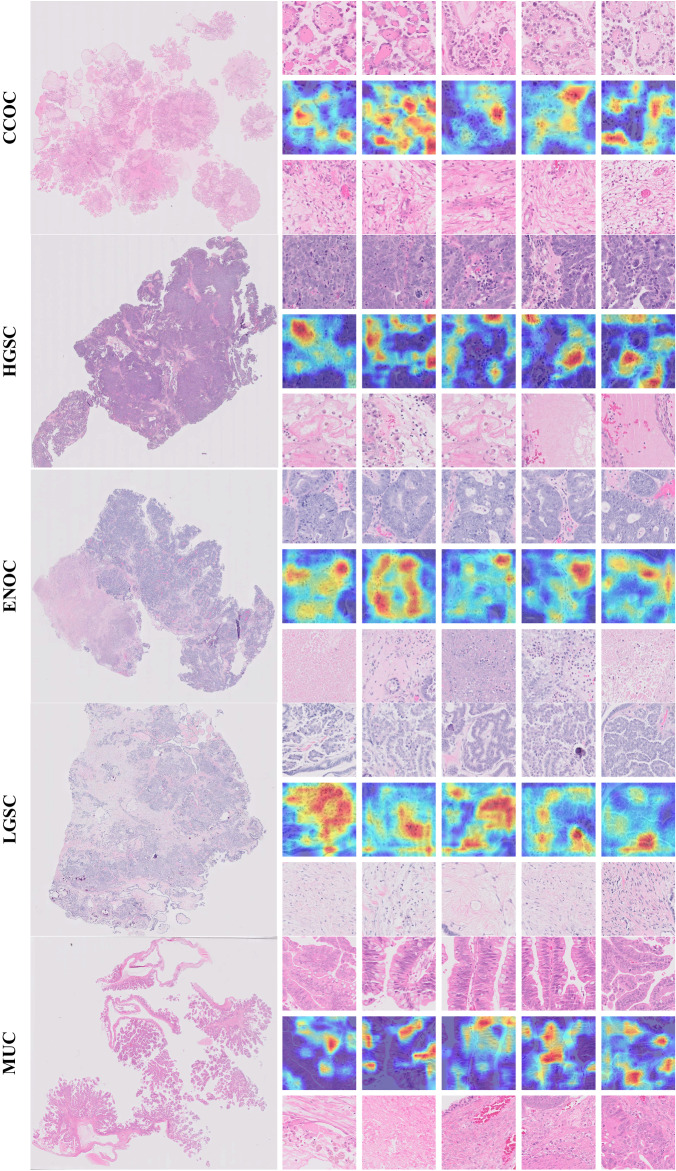
Examples of ovarian cancer subtype classification for five major histotypes (i.e., HGSC, ENOC, CCOC, LGSC, and MUC). One sample from each histotype is shown. The left column shows the whole-slide image and the right column shows (top) the top five patches; (middle) the corresponding attention/importance heatmap highlighting the most significant regions in the image for predicting histotype; and (bottom) the bottom five patches.

Figure 9 identifies relevant histological features for ovarian epithelial tumor histotype classification. The figure shows representative samples for each ovarian cancer histotype, displaying (1) the top five patches for subtype classification (first row for each sample), (2) corresponding importance heatmaps from the top five patches (second row for each sample), and (3) the bottom five patches (third row for each sample). To visualize the key regions of the image associated with the relevant diagnosis, we used the class-discriminative localization method proposed in ref. ^56^. To assign importance values to each neuron in the target category (i.e., the ground truth label), this method utilizes gradient information flowing into the last convolutional layer of the network. As a result, this method uses fine-grained details in an image to localize the target category. We applied this method to the top five patches. This enabled us to visualize the areas of the input image that strongly influence the output of the classification model.

The top five patches selected by the method contained subtype-specific histologic features including tumor epithelium, while the bottom five patches primarily encompassed nonspecific stromal or necrotic areas (Fig. 9). For example, the most discriminative areas within the top five patches for clear cell carcinomas contained eosinophilic hyaline globules, a typical feature of the clear cell histotype^57^. This finding highlights that the discriminatory power of the method is not limited to just the cytoarchitectural features of tumor cells, but also those of characteristic stromal elements.

**Fig. 10:**
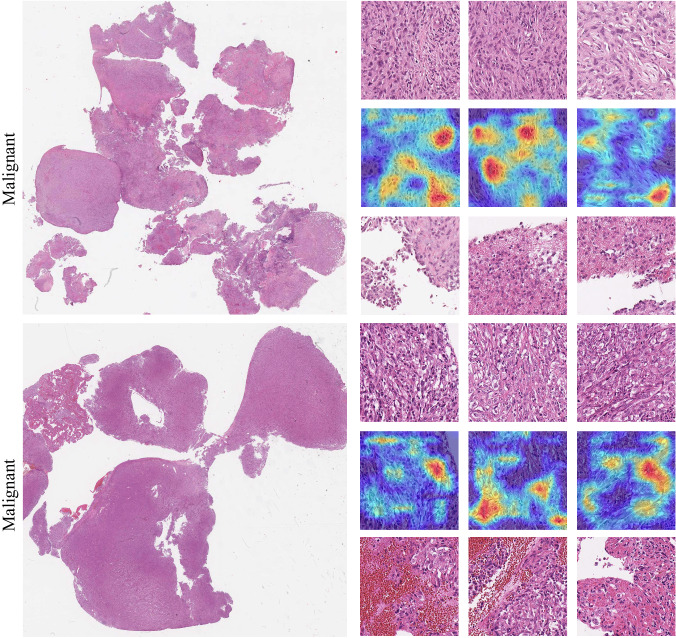
Examples of pleural malignant classification. The left column shows two selected whole-slide images and the right column shows (top) the top three patches; (middle) the corresponding attention/importance heatmap highlighting the most significant regions in the image for predicting histotype; and (bottom) the bottom three patches.

**Fig. 11:**
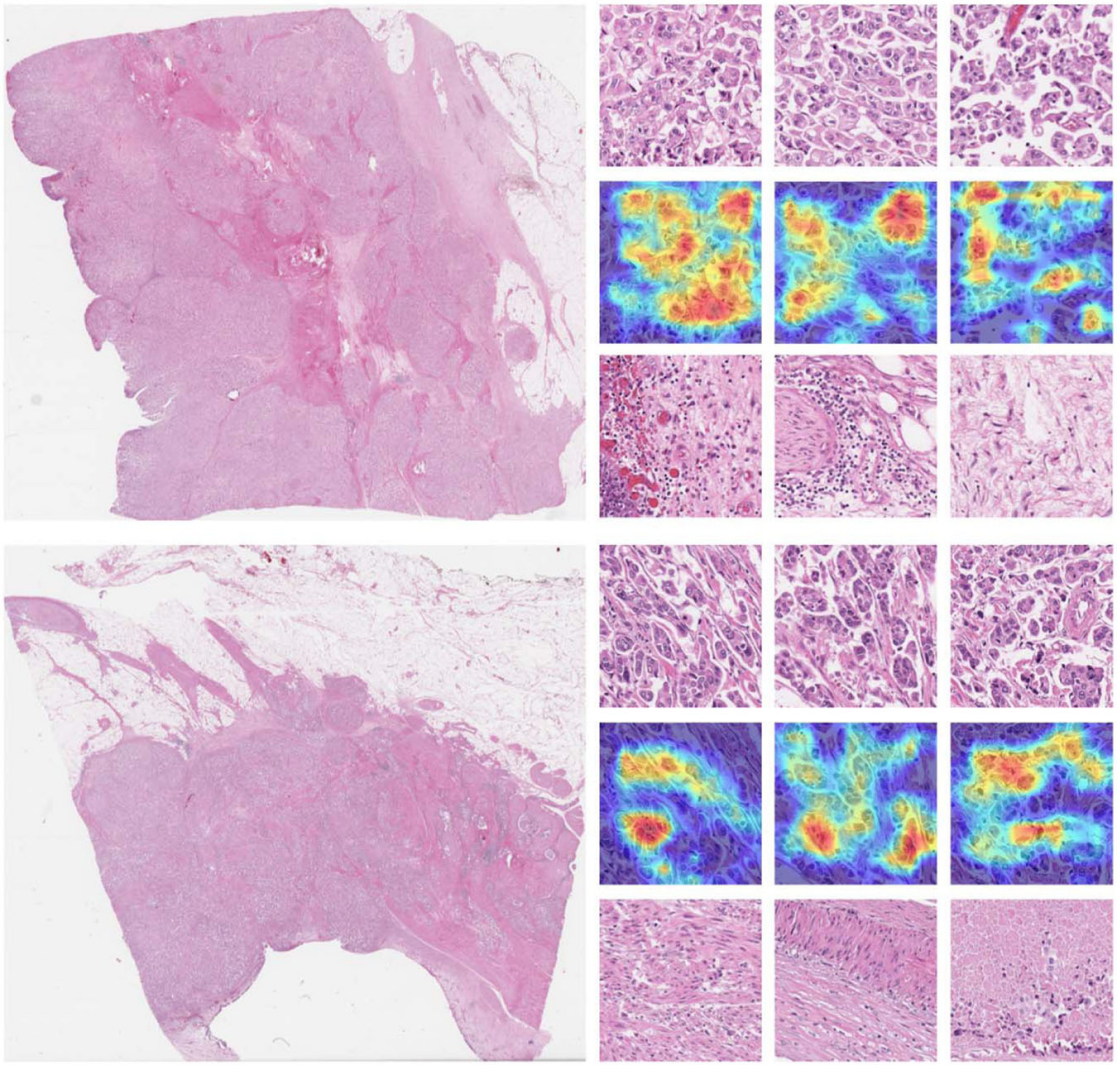
Examples of micro-papillary classification. The left column shows two selected whole-slide images and the right column shows (top) the top three patches; (middle) the corresponding attention/importance heatmap highlighting the most significant regions in the image for predicting histotype; and (bottom) the bottom three patches.

This visualization is also available for representative malignant cases within the Pleural and Bladder cancer datasets (Figs. 10 and 11). In the pleural cancer cases, the top three patches showed high cellularity with densely packed spindle cells, while the low-ranked patches were much more paucicellular and featured areas of collagen. In bladder cancer, the top three patches selected by the method contained subtype-specific histologic features including tumor epithelium, while the bottom three patches primarily encompassed nonspecific stromal or necrotic areas. For example, the most discriminative areas within the top three patches demonstrate the presence of multiple tumor cell clusters within the same lacuna with peripherally oriented nuclei, a typical feature of micropapillary urothelial carcinoma^58^.

## Discussion

Domain shift in histopathology data can pose significant difficulties for deep learning-based classifiers, as models trained on data from a single center may overfit to that data and fail to generalize well to external datasets. To overcome this challenge, adversarial domain adaptation networks have been employed, however, these networks tend to decrease the discriminability of the learned features and do not fully utilize the knowledge transferability of the target domain. To address these shortcomings, we proposed an approach referred to as AIDA, which enhances the adversarial domain adaptation network using the frequency domain information through an FFT-Enhancer module. By integrating the color space of target domain samples into the label prediction loss, our approach effectively addressed the challenge of overfitting the network to the source domain. This integration yielded significant benefits, as the network demonstrated enhanced generalization capabilities, enabling it to more accurately classify the target domain. Consequently, our approach surpassed the limitations of previous methods by improving the network’s discriminability for both the source and target domains.

The proposed AIDA framework was implemented on four datasets related to ovarian, pleural, bladder, and breast cancers. In all datasets, AIDA demonstrated superior performance in target domains based on different metrics including balanced accuracy, Cohen’s Kappa, F1-score, and AUC, compared to the Base, HED, Macenko, CTransPath, CNorm, and ADA. Furthermore, AIDA exhibited superior performance compared to other methods in the source domain of Ovarian and Pleural datasets. Additionally, the incorporation of the FFT-Enhancer exhibited a noticeable improvement in the performance of the Base-FFT model, outperforming the Base model. These results underscore the importance of integrating the FFT-Enhancer module in the model architecture to enhance knowledge transfer between domains, resulting in more robust and reliable models for real-world applications.

Broadly speaking, there are two main methods for addressing domain shift: domain adaptation and domain generalization. Domain adaptation typically necessitates access to both source and target data during training, albeit relying solely on labeled data from the source domain. Conversely, domain generalization offers the advantage of not requiring target data for training. However, it is essential to acknowledge that domain generalization methods are not infallible and may encounter challenges in certain scenarios. Techniques like HED, Macenko, CTransPath, and CNorm are categorized as domain generalization approaches, yet AIDA outperformed each of them, showcasing its superior capability in addressing domain shift when compared to conventional domain generalization methods. This reinforces the significance of our proposed framework in enhancing knowledge transfer between domains and producing more robust and reliable models for real-world applications.

Furthermore, we evaluated the utility and value of different convolutional layers as feature extractors in AIDA and showed that utilizing the fourth convolutional block as input features for the domain discriminator resulted in the most generalized performance. As opposed to the fourth convolutional block, the second and third blocks generate lower-level features with smaller local receptive fields which caused inferior performance when used for the domain classifier. We hypothesize that, when using labeled data of the source domain for model training, the fifth convolutional block learns semantic information and derives features that are highly specific to the source domain classification, and therefore, such a strategy did not yield superior performance compared to the fourth layer.

Our experiments demonstrated that AIDA consistently outperformed ADA across various backbone architectures. Furthermore, when utilizing foundation models as the backbone with domain-specific pre-trained weights instead of ImageNet weights, AIDA still exhibited superior performance compared to ADA. We compared the performance of a foundation model trained on a substantial number of histopathology slides with AIDA fine-tuned using this foundation model as the backbone. The results indicated that for three out of four datasets, fine-tuning AIDA with the foundation model and domain-specific pre-trained weights yielded better performance than using the foundation model alone. This suggests that while foundation models provide strong performance, AIDA can further enhance their effectiveness. Additionally, AIDA employing a backbone with domain-specific pre-trained weights achieved superior performance compared to AIDA using a backbone with ImageNet pre-trained weights in two datasets. This demonstrates that AIDA can also benefit from domain-specific pre-trained weights. For all four datasets, training AIDA with the foundation model as the backbone yielded better results without using any augmentation methods, a scenario in which ADA did not perform well. This suggests that domain-specific pre-trained weights facilitate adaptation to various augmentations. Consequently, without augmentations, FFT-Enhancer is likely to encourage the feature extraction process to focus more on tumor morphology and shape.

The t-SNE-based visualizations demonstrated that the AIDA model improved the discriminability of different subtypes in the feature representation space compared to the Base and CNorm models. The learned features by AIDA exhibited less overlap and consequently, more discrimination between the subtypes. Furthermore, our investigation reveals a prominent concurrence between the tumor annotations provided by the pathologist and the corresponding heatmaps generated by AIDA method. This compelling alignment serves as conclusive evidence, substantiating the efficacy of our proposed approach in accurately localizing the tumor areas.

Moreover, our study revealed that the top patches of slides exhibited subtype-specific histologic features, such as tumor epithelium, while the bottom five patches predominantly contained nonspecific stromal or necrotic areas. We employed a class-discriminative localization method to identify and highlight the relevant histological features on these patches. Subsequent reviews by pathologists confirmed that the highlighted areas contained subtype-specific tumor information, providing evidence for the usefulness and reliability of our proposed method in identifying and localizing relevant histological features.

It is important to highlight that, compared to the Pleural, Bladder, and Breast datasets, the proposed AIDA led to greater performance improvements when applied to the Ovarian dataset. This could be attributed to several factors. Firstly, the Ovarian dataset contains a significantly larger number of slides (>1000) than the other datasets, potentially resulting in more color variations. Additionally, it should be noted that the classification task for the Pleural, Bladder, and Breast dataset involves the discrimination between samples from two subtypes, while the Ovarian cancer dataset requires the classification of five distinct subtypes, making it a more challenging task.

Our experimental results demonstrated the effectiveness of AIDA in achieving promising performance across four large datasets encompassing diverse cancer types. However, there are several avenues for future research that can contribute to the advancement of this work. Firstly, it is important to validate the generalizability of AIDA by conducting experiments on other large datasets. Moreover, the applicability of AIDA can be extended beyond cancer subtype classification to other histopathology tasks. Tasks such as tumor segmentation, mitotic figure detection, or cancer grading can benefit from the proposed method. In the future, exploring alternative backbone architectures can be an intriguing direction for future investigation.

## Methods

### Ethics statement

The Declaration of Helsinki and the International Ethical Guidelines for Biomedical Research Involving Human Subjects were strictly adhered throughout the course of this study. All protocols for this study, including the waiver of consent, have been approved by the University of British Columbia/BC Cancer Research Ethics Board. Participants did not receive compensation.

### Data

In our experiments, we used datasets related to four different cancers: ovarian, pleural, bladder, and breast (Table 1). The Ovarian dataset contains WSIs of the five major histological subtypes of epithelial ovarian carcinomas, namely clear cell ovarian carcinoma (CCOC), endometrioid carcinoma (ENOC), high-grade serous carcinoma (HGSC), low-grade serous carcinoma (LGSC), and mucinous carcinoma (MUC). The source and target ovarian cancer datasets are processed and digitized in two different centers and include 1053 and 60 WSIs from 523 and 60 patients, respectively.

The Pleural dataset consists of benign pleural tissue and malignant mesothelioma slides from two centers. The source dataset includes 194 WSIs (128 patients) and the target dataset contains 53 WSIs (53 patients). The Bladder dataset is comprised of micropapillary carcinoma (MPC) and conventional urothelial carcinoma (UCC) slides from multiple hospitals across British Columbia. The source dataset encompasses 262 WSIs from 86 patients belonging to the source domain. On the other hand, the target domain includes 160 slides from 72 patients.

For the Breast dataset, we employed the publicly available ICIAR-2018 dataset as the source domain and the BreaKHis dataset as the target domain. The source domain consists of 400 breast biopsy images obtained from 257 patients, representing four subtypes: normal, benign, in situ carcinoma, and invasive carcinoma. In contrast, the target domain comprises 7909 small biopsy images from 82 patients, categorized into benign and malignant breast tissue. To facilitate classification, we consolidated the four subtypes in the source domain into two overarching categories: benign and malignant. Notably, both datasets are provided in patch format rather than whole slide images.

Supplementary Fig. 2 shows representative patches from the source and target domains associated with Ovarian (first row), Pleural (second row) datasets, and Bladder (third row) datasets; highlighting the variability in color profiles.

### Problem definitions

We denote ${{{\mathcal{{D}}}_{S}}}$ as the set of patches of the source domain which contains $\{({x}_{i}^{s},{y}^{s})\,| i\in \{1,\ldots ,{N}_{s}\},s\in \{1,2,\ldots ,S\}\}$, where *N*_*s*_ is the number of patches of the *s*^th^ slide, *S* is the number of slides in source domain, ${x}_{i}^{s}$ is the *i*^th^ patch of the *s*^th^ slide, and *y*^*s*^ is the patch label which corresponds to the slide label. The patches extracted from the target domain, ${{{\mathcal{{D}}}_{T}}}$, consists of $\{{x}_{i}^{t}| i\in \{1,\ldots ,{N}_{t}\},t\in \{1,\ldots ,T\}\}$, where *N*_*t*_ is the number of patches of the *t*^th^ slide, *T* is the number of slides in target domain, and ${x}_{i}^{t}$ is the *i*^th^ patch of the *t*^th^ slide of the target domain. Our goal is to learn a feature representation in a shared space that is discriminative for label prediction while being insensitive to the domain. To do that, we propose AIDA (Fig. 1) by integrating the FFT-Enhancer module in an adversarial neural network.

### Fourier-based enhancer (FFT-enhancer) module

In the frequency domain, the predominant effect of domain shift is amplitude changes, while the phase information is nearly identical (Supplementary Fig. 3). In this paper, we propose integrating the adversarial network with the FFT-Enhancer.

Consider $({x}_{i}^{s},{y}_{i}^{s})\in {{{\mathcal{{D}}}_{S}}}$ and ${x}_{j}^{t}\in {{{\mathcal{{D}}}_{T}}}$ as a sample from the source and target data, respectively. The Fourier transformation of these samples are

1 $$\begin{array}{ll}{X}_{i}^{s}\,=\,{{{{\mathcal{A}}}}}_{i}^{s}\otimes {e}^{{{{\bf{j}}}}.{{{{\mathcal{P}}}}}_{i}^{s}},\\ {X}_{j}^{t}\,=\,{{{{\mathcal{A}}}}}_{j}^{t}\otimes {e}^{{{{\bf{j}}}}.{{{{\mathcal{P}}}}}_{j}^{t}},\end{array}$$

where ${X}_{i}^{s}$ is the Fourier domain of the *i*^th^ patch of the *s*^th^ slide of the source domain, and ${X}_{j}^{t}$ is the Fourier domain of the *j*^th^ patch of the *t*^th^ slide of the target domain. Additionally, $({{{{\mathcal{A}}}}}_{i}^{s},{{{{\mathcal{P}}}}}_{i}^{s})$ and $({{{{\mathcal{A}}}}}_{j}^{t},{{{{\mathcal{P}}}}}_{j}^{t})$ show the amplitude and phase spectrum of ${x}_{i}^{s}$ and ${x}_{j}^{t}$, respectively, and ⊗ represents the element-wise multiplication. Through the FFT-Enhancer, we produce a new sample of

2 $$Z({x}_{s}^{i},{x}_{t}^{j})=iDFT\left({{{{\mathcal{A}}}}}_{j}^{t}\otimes {e}^{{{{\bf{j}}}}.{{{{\mathcal{P}}}}}_{i}^{s}}\right),$$

by substituting the amplitude of the source patch with that of the target patch. *iDFT* is the inverse of Discrete Fourier Transform (DFT). To enable the network to pay more attention to the phase information and be insensitive to amplitude (i.e., color variations), the new image is produced by the recombination of amplitude information from the target sample and the phase from the source sample. The resulting image serves as a representative image from the source dataset during the training phase. Supplementary Fig. 3 showcases multiple illustrative examples of this process.

### Adversarial network

There are three components in the adversarial network: a feature extractor, a label predictor, and a domain classifier (Fig. 1). The first component derives feature embeddings from the extracted patches, which are subsequently passed to the label predictor and domain classifier as the input data. The feature extraction step is shared among the target and source datasets. We train the label predictor on the source data, while the domain classifier uses both the source and target features. Different backbones including convolutional and fully connected layers can be used as the feature extractor. The feature extractor learns the function ${G}_{f}(x,{\theta }_{f}):x\in \{{{{\mathcal{{D}}}_{S}}}\cup {{{\mathcal{{D}}}_{T}}}\}\to {{\mathbb{R}}}^{m}$, where *x* can be a patch from both source (${{{\mathcal{{D}}}_{S}}}$) and target (${{{\mathcal{{D}}}_{T}}}$) data, ${{\mathbb{R}}}^{m}$ is the learned *m* dimensional feature representation, and *θ*_*f*_ is the set of parameters learned through the feature extractor. This function maps the input image from both source and target to an *m* dimensional feature vector. The extracted features of the source data are then passed to the label predictor. The label predictor is a function ${G}_{y}(({G}_{f}({x}_{i}^{s},{\theta }_{f}),{y}^{s}),{\theta }_{y}):{{\mathbb{R}}}^{m}\to \{1,2,...,Y\}$, where ${{\mathbb{R}}}^{m}$ is the extracted features, *y*^*s*^ is the patch label (i.e., the label of the *s*^th^ slide), *θ*_*y*_ is the parameters of label predictor, and {1, 2, . . . , *Y*} is the set of labels we have for the source domain. This function predicts a label for the features extracted from an input sample of the source domain. In a supervised manner, we adopt the cross-entropy loss to optimize the performance of the label predictor as

3 $${{{{\mathcal{L}}}}}_{p}=-\frac{1}{{\Sigma }_{s = 1}^{S}{N}_{s}}{\Sigma }_{s = 1}^{S}{\Sigma }_{i = 1}^{{N}_{s}}\,{y}^{s}\,\log \left[{G}_{y}\left(\left({G}_{f}({x}_{i}^{s},{\theta }_{f}),{y}^{s}\right),{\theta }_{y}\right)\right],$$

where ${{{{\mathcal{L}}}}}_{p}$ is the label predictor loss computed for the source patches. Following the idea of generative adversarial network (GAN) loss^59^, adversarial training is employed for the domain classification. We pass the learned feature representation of both source and target data to a discriminator as a domain regularizer. The domain discriminator ${G}_{d}(({G}_{f}({x}_{i},{\theta }_{f}),{d}_{i}),{\theta }_{d}):{{\mathbb{R}}}^{m}\to \{0,1\}$ maps the extracted features of $\{{x}_{i}\in {{{\mathcal{{D}}}_{S}}}\cup {{{\mathcal{{D}}}_{T}}}\}$ into a binary domain label of {0, 1} by learning *θ*_*d*_ as the set of parameters of the discriminator. The domain label for the patches *x*_*i*_ is represented by *d*_*i*_ ∈ {0, 1} in which *d*_*i*_ = 1 and 0 are the labels for the source and target domains, respectively. The whole network is learned in an adversarial manner to purposely misdirect the domain discriminator so that the domain discriminator cannot distinguish between the two feature spaces. The adversarial loss of the discriminator is computed as

4 $$\begin{array}{ll}{{{{\mathcal{L}}}}}_{adv}\,=\,-\frac{1}{{\Sigma }_{s = 1}^{S}{N}_{s}}{\Sigma }_{s = 1}^{S}{\Sigma }_{i = 1}^{{N}_{s}}\,\log \left[{G}_{d}\left({G}_{f}({x}_{i}^{s},{\theta }_{f}),{d}_{i}=1\right)\right]\\ \qquad\quad\,\,-\frac{1}{{\Sigma }_{t = 1}^{T}{N}_{t}}{\Sigma }_{t = 1}^{T}{\Sigma }_{i = 1}^{{N}_{t}}\,\log \left[1-{G}_{d}\left({G}_{f}({x}_{i}^{t},{\theta }_{f}),{d}_{i}=0\right)\right],\end{array}$$

where ${{{{\mathcal{L}}}}}_{adv}$ is the adversarial loss for domain classification. A gradient reversal layer connects the discriminator and the feature extractor and multiplies the gradient by a negative constant during backpropagation. We define ${{{\mathcal{L}}}}={{{{\mathcal{L}}}}}_{p}+{{{{\mathcal{L}}}}}_{adv}$ as the training loss.

### Slide classification

The proposed pipeline is applied to patches extracted from a given slide. To achieve slide-level classification, We employ VLAD encoding^45^, a Multiple Instance Learning (MIL)-based aggregation function that is used to produce slide-level representation by using features of the patches within the slide. Details of VLAD encoding can be found in ref. ^45^. After practicing this step, a Support Vector Machine (SVM) classifier is trained to assign the label for a given slide.

### Experimental design

WSIs are large gigapixel images with resolutions typically exceeding 100,000 × 100,000 pixels and present a high degree of morphological variance, as well as containing a variety of artifacts. These conditions make it impossible to directly apply conventional deep networks. For the Ovarian, Pleural, and Bladder datasets, whole slide images (WSIs) serve as the input data. For computational tractability, we selected smaller regions from a WSI (referred to as patches) to train and build our model. More specifically, we extracted 150 patches per slide, with 1024 × 1024 pixels resolution. For the source domain of the Ovarian and Pleural datasets, the patches were extracted from the pathologist-annotated areas (containing tumor tissue) while we randomly extracted patches for the target domain. This random extraction approach was adopted for the target domain due to the consideration of target data as unsupervised, with the assumption that no prior information is available for this dataset. For the Bladder dataset, patches were extracted randomly from both the source and target datasets because pathologists’ annotations were not accessible. The patches were then resized to 512 × 512 pixels, ensuring a standardized magnification of 20X. We employed a three-fold cross-validation strategy to evaluate the performance of the model on the source domain. The resulting three cross-validation models were evaluated on the target domain. Both domains of the breast dataset consisted of patches rather than WSIs of the tissue. In order to ensure compatibility between domains within this dataset, we extracted patches with a resolution of 230 × 230 pixels at 20X magnification.

For each experimental iteration, the complete pipeline underwent training with five distinct random seeds, and the average performance across three cross-validation runs was reported for both the source and target domains. The networks were trained with a learning rate of 1e−4, utilizing a batch size of 256 patches and a predefined maximum number of epochs set to 20. Unless otherwise specified, all experiments employed standard augmentation techniques such as random rotation, flipping, and color jittering. The Adam optimizer^60^ was employed during the network optimization process. To mitigate overfitting, the training procedure was halted if no improvement in network loss was observed over ten consecutive epochs. Furthermore, VLAD encoding required the development of a codebook, with a predetermined size of *k* = 4 for two datasets: Ovarian and Pleural. However, for the Breast and Bladder dataset, a smaller size of *k* = 1 was found to be optimal based on our experimental findings. The selection of the codebook size was guided by the model’s performance on the validation set within the source domain. Supplementary Fig. 4 presents the balanced accuracy of the model across various *k* values. Various performance metrics were employed for comprehensive comparative analysis, including balanced accuracy, Cohen’s Kappa, F1 Score, and AUC.

### Underlying network architectures

The baseline architecture employed in our study was ResNet18^61^. To test the impact of solely FFT-Enhancer on the output, we trained both the baseline and adversarial networks with and without this module. It’s important to note that while the FFT-Enhancer can enhance images, it’s not always perfect, and there may be instances of noise artifacts in the output image. To assess its impact on the model, we experimented with different probabilities of applying the FFT-Enhancer during training for both AIDA and Base-FFT. Optimal results were achieved with probabilities ranging from 40% to 60% across all datasets. Decreasing the probability below 40% led to a drop in the models’ balanced accuracy, as insufficient staining information from the target domain was utilized during training. Conversely, applying the FFT-Enhancer more than 60% resulted in noise artifacts that hindered the network’s performance.

To ensure a fair and equitable comparison, unless stated otherwise, we used ResNet18 as the backbone architecture for feature extraction in all experiments. The domain classifier itself consisted of three fully connected layers with dimensions of ${{\mathbb{R}}}^{m}\times 32\times 32$, 256, and 100 neurons, respectively. Furthermore, it is pertinent to highlight that the pre-trained weights of ImageNet were consistently employed for the backbone models in all conducted experiments.

### Comparative analysis of training times and computational resources: base vs. AIDA

In our experiments, all methods were executed on an NVIDIA GeForce RTX 3090 GPU, with 24 GB of GDDR6X memory. Given that the Base, Base-FFT, Macenko, HED, and CNorm methods all shared identical architectures, and ADA and AIDA exhibited similar structures, we will focus solely on comparing the time analysis between the Base and AIDA. This comparison will provide insights into their respective training times and utilization of computational resources, as outlined in Supplementary Table 7.

The Base and AIDA differ in terms of model complexity, with AIDA being slightly more intricate than Base. This difference in complexity contributes to variations in their training times. However, during inference, where both networks leverage the same backbone, feature extraction, and multiple instance learning processes require a comparable amount of time for both models. Consequently, the longer training duration of AIDA does not directly correlate with extended inference time or computational overhead during testing. AIDA’s superior performance in cancer subtype classification justifies its lengthier training period. The heightened model complexity empowers AIDA to capture intricate patterns and relationships within the data, thereby enhancing classification accuracy. Consequently, despite AIDA’s larger parameter count and slightly prolonged training time, it is crucial to underscore the primary objective of achieving accurate cancer subtype classification.

### Statistical assessment

The non-parametric version of the paired T-test, namely the Wilcoxon signed-rank test (two-sided), was employed to assess the statistical significance of the difference between the balanced accuracy achieved by different methods. Throughout all experiments, *p* < 0.05 was regarded as the significance level.

### Supplementary information

Supplementary File

## Data Availability

The ICIAR-2018 whole slide images and their respective labels can be accessed from the BACH grand challenge page at (https://iciar2018-challenge.grand-challenge.org/Dataset/). Additionally, the BreaKHis patches are publicly accessible via the Kaggle challenge page at (https://www.kaggle.com/datasets/ambarish/breakhis). For queries concerning the academic utilization of our confidential data, kindly direct your communication to the corresponding author. We will carefully evaluate any pertinent intellectual property rights or obligations regarding patient confidentiality, adhering to the protocols established by our institution and department. Please be advised that processing such requests may necessitate the execution of a material transfer agreement.

## Code availability

The code used in this manuscript will be publicly available at https://github.com/AIMLab-UBC/AIDA.git.

## References

[1] Farahmand, S. et al. Deep learning trained on hematoxylin and eosin tumor region of interest predicts her2 status and trastuzumab treatment response in her2+ breast cancer. *Mod. Pathol.* **35**, 44–51 (2022). 34493825 10.1038/s41379-021-00911-w PMC10221954

[2] Xie, W. et al. Prostate cancer risk stratification via nondestructive 3d pathology with deep learning–assisted gland analysis. *Cancer Res.* **82**, 334–345 (2022). 34853071 10.1158/0008-5472.CAN-21-2843 PMC8803395

[3] Flinner, N. et al. Deep learning based on hematoxylin-eosin staining outperforms immunohistochemistry in predicting molecular subtypes of gastric adenocarcinoma. *J. Pathol.* **257**, 218–226 (2022). 10.1002/path.5879 35119111

[4] Bulten, W. et al. Artificial intelligence for diagnosis and gleason grading of prostate cancer: the panda challenge. *Nat. Med.* **28**, 154–163 (2022). 35027755 10.1038/s41591-021-01620-2 PMC8799467

[5] Zheng, X. et al. A deep learning model and human-machine fusion for prediction of ebv-associated gastric cancer from histopathology. *Nat. Commun.* **13**, 2790 (2022). 35589792 10.1038/s41467-022-30459-5 PMC9120175

[6] Dolezal, J. M. et al. Uncertainty-informed deep learning models enable high-confidence predictions for digital histopathology. *Nat. Commun.* **13**, 6572 (2022). 36323656 10.1038/s41467-022-34025-x PMC9630455

[7] Shamai, G. et al. Deep learning-based image analysis predicts pd-l1 status from h&e-stained histopathology images in breast cancer. *Nat. Commun.* **13**, 6753 (2022). 36347854 10.1038/s41467-022-34275-9 PMC9643479

[8] Darbandsari, A. et al. AI-based histopathology image analysis reveals a distinct subset of endometrial cancers. *Nat. Commun.* **15**, 4973 (2024). 38926357 10.1038/s41467-024-49017-2 PMC11208496

[9] Laleh, N. G. et al. Benchmarking weakly-supervised deep learning pipelines for whole slide classification in computational pathology. *Med. Image Anal.* **79**, 102474 (2022). 35588568 10.1016/j.media.2022.102474

[10] Foersch, S. et al. Multistain deep learning for prediction of prognosis and therapy response in colorectal cancer. *Nat. Med.* **29**, 430–439 (2023). 10.1038/s41591-022-02134-1 36624314

[11] Tolkach, Y., Dohmgörgen, T., Toma, M. & Kristiansen, G. High-accuracy prostate cancer pathology using deep learning. *Nat. Mach. Intell.* **2**, 411–418 (2020). 10.1038/s42256-020-0200-7

[12] Boschman, J. et al. The utility of color normalization for AI-based diagnosis of hematoxylin and eosin-stained pathology images. *J. Pathol.* **256**, 15–24 (2021). 34543435 10.1002/path.5797

[13] Ren, J., Hacihaliloglu, I., Singer, E. A., Foran, D. J. & Qi, X. Unsupervised domain adaptation for classification of histopathology whole-slide images. *Front. Bioeng. Biotechnol.* **7**, 102 (2019). 31158269 10.3389/fbioe.2019.00102 PMC6529804

[14] Bejnordi, B. E. et al. Stain specific standardization of whole-slide histopathological images. *IEEE Trans. Med. imaging* **35**, 404–415 (2015). 26353368 10.1109/TMI.2015.2476509

[15] Ganin, Y. et al. Domain-adversarial training of neural networks. *J. Mach. Learn. Res.* **17**, 2096–2030 (2016).

[16] Sikaroudi, M., Rahnamayan, S. & Tizhoosh, H. R. Hospital-agnostic image representation learning in digital pathology. In *2022 44th Annual International Conference of the IEEE Engineering in Medicine & Biology Society (EMBC)*, 3055–3058 (IEEE, 2022). 10.1109/EMBC48229.2022.9871198 36086646

[17] Cheng, B., Liu, M., Zhang, D., Munsell, B. C. & Shen, D. Domain transfer learning for mci conversion prediction. *IEEE Trans. Biomed. Eng.* **62**, 1805–1817 (2015). 25751861 10.1109/TBME.2015.2404809 PMC4474791

[18] Gu, Y., Ge, Z., Bonnington, C. P. & Zhou, J. Progressive transfer learning and adversarial domain adaptation for cross-domain skin disease classification. *IEEE J. Biomed. health Inform.* **24**, 1379–1393 (2019). 31545748 10.1109/JBHI.2019.2942429

[19] Ruiz, A. et al. Pathological image analysis using the gpu: Stroma classification for neuroblastoma. In *2007 IEEE International Conference on Bioinformatics and Biomedicine (BIBM 2007)*, 78–88 (IEEE, 2007).

[20] Jafari-Khouzani, K. & Soltanian-Zadeh, H. Multiwavelet grading of pathological images of prostate. *IEEE Trans. Biomed. Eng.* **50**, 697–704 (2003). 12814236 10.1109/TBME.2003.812194

[21] Tellez, D. et al. Whole-slide mitosis detection in h&e breast histology using phh3 as a reference to train distilled stain-invariant convolutional networks. *IEEE Trans. Med. imaging* **37**, 2126–2136 (2018). 29994086 10.1109/TMI.2018.2820199

[22] Tellez, D. et al. Quantifying the effects of data augmentation and stain color normalization in convolutional neural networks for computational pathology. *Med. image Anal.* **58**, 101544 (2019). 31466046 10.1016/j.media.2019.101544

[23] Bug, D. et al. Context-based normalization of histological stains using deep convolutional features. In *Deep Learning in Medical Image Analysis and Multimodal Learning for Clinical Decision Support: Third International Workshop, DLMIA 2017, and 7th International Workshop, ML-CDS 2017, Held in Conjunction with MICCAI 2017, Québec City, QC, Canada, September 14, Proceedings 3*, 135–142 (Springer, 2017).

[24] Reinhard, E., Adhikhmin, M., Gooch, B. & Shirley, P. Color transfer between images. *IEEE Computer Graph. Appl.* **21**, 34–41 (2001). 10.1109/38.946629

[25] Koch, V. et al. Noise transfer for unsupervised domain adaptation of retinal oct images. In *International Conference on Medical Image Computing and Computer-Assisted Intervention*, 699–708 (Springer, 2022).

[26] Macenko, M. et al. A method for normalizing histology slides for quantitative analysis. In *2009 IEEE international symposium on biomedical imaging: from nano to macro*, 1107–1110 (IEEE, 2009).

[27] Tabesh, A. et al. Multifeature prostate cancer diagnosis and gleason grading of histological images. *IEEE Trans. Med. imaging* **26**, 1366–1378 (2007). 17948727 10.1109/TMI.2007.898536

[28] Diao, P., Pai, A., Igel, C. & Krag, C. H. Histogram-based unsupervised domain adaptation for medical image classification. In *International Conference on Medical Image Computing and Computer-Assisted Intervention*, 755–764 (Springer, 2022).

[29] Zhu, J.-Y., Park, T., Isola, P. & Efros, A. A. Unpaired image-to-image translation using cycle-consistent adversarial networks. In *Proceedings of the IEEE international conference on computer vision*, 2223–2232 (IEEE, 2017).

[30] Runz, M. et al. Normalization of he-stained histological images using cycle consistent generative adversarial networks. *Diagnostic Pathol.* **16**, 1–10 (2021). 10.1186/s13000-021-01126-y PMC8349020 34362386

[31] Zhou, Y. et al. Multi-site cross-organ calibrated deep learning (muscld): Automated diagnosis of non-melanoma skin cancer. *Med. Image Anal.* **84**, 102702 (2023). 36516556 10.1016/j.media.2022.102702 PMC9825103

[32] Ye, H.-L. & Wang, D.-H. Stain-adaptive self-supervised learning for histopathology image analysis. Preprint at https://arxiv.org/abs/2208.04017 (2022).

[33] Tiard, A. et al. Stain-invariant self supervised learning for histopathology image analysis. Preprint at https://arxiv.org/abs/2211.07590 (2022).

[34] Howard, F. M. et al. The impact of site-specific digital histology signatures on deep learning model accuracy and bias. *Nat. Commun.* **12**, 1–13 (2021). 34285218 10.1038/s41467-021-24698-1 PMC8292530

[35] Lafarge, M. W., Pluim, J. P., Eppenhof, K. A., Moeskops, P. & Veta, M. Domain-adversarial neural networks to address the appearance variability of histopathology images. In *Deep learning in medical image analysis and multimodal learning for clinical decision support*, 83–91 (Springer, 2017).

[36] Otálora, S., Atzori, M., Andrearczyk, V., Khan, A. & Müller, H. Staining invariant features for improving generalization of deep convolutional neural networks in computational pathology. *Front. Bioeng. Biotechnol.* **7**, 198 (2019). 10.3389/fbioe.2019.00198 PMC6716536 31508414

[37] Wang, S. & Zhang, L. Self-adaptive re-weighted adversarial domain adaptation. *In Proceedings of the Twenty-Ninth International Conference on Artificial Intelligence,* (2021).

[38] Xiao, T., Fan, C., Liu, P. & Liu, H. Simultaneously improve transferability and discriminability for adversarial domain adaptation. *Entropy* **24**, 44 (2022). 10.3390/e24010044 PMC8775043 35052070

[39] Mehra, A., Kailkhura, B., Chen, P.-Y. & Hamm, J. Understanding the limits of unsupervised domain adaptation via data poisoning. *Adv. Neural Inf. Process. Syst.* **34**, 17347–17359 (2021).

[40] Chen, X., Wang, S., Long, M. & Wang, J. Transferability vs. discriminability: Batch spectral penalization for adversarial domain adaptation. In *International conference on machine learning*, 1081–1090 (PMLR, 2019).

[41] Koohbanani, N. A., Unnikrishnan, B., Khurram, S. A., Krishnaswamy, P. & Rajpoot, N. Self-path: Self-supervision for classification of pathology images with limited annotations. *IEEE Trans. Med. Imaging* **40**, 2845–2856 (2021). 33523807 10.1109/TMI.2021.3056023

[42] Abbet, C. et al. Self-rule to adapt: Learning generalized features from sparsely-labeled data using unsupervised domain adaptation for colorectal cancer tissue phenotyping. In *Medical Imaging with Deep Learning* (PMLR, 2021).

[43] Wang, H., Wu, X., Huang, Z. & Xing, E. P. High-frequency component helps explain the generalization of convolutional neural networks. In *Proceedings of the IEEE/CVF Conference on Computer Vision and Pattern Recognition*, 8684–8694 (IEEE, 2020).

[44] Chen, G. et al. Amplitude-phase recombination: Rethinking robustness of convolutional neural networks in frequency domain. In *Proceedings of the IEEE/CVF International Conference on Computer Vision*, 458–467 (IEEE, 2021).

[45] Jégou, H., Douze, M., Schmid, C. & Pérez, P. Aggregating local descriptors into a compact image representation. In *2010 IEEE computer society conference on computer vision and pattern recognition*, 3304-3311 (IEEE, 2010).

[46] Wang, X. et al. Transformer-based unsupervised contrastive learning for histopathological image classification. *Med. Image Anal.* **81**, 102559 (2022). 35952419 10.1016/j.media.2022.102559

[47] Aresta, G. et al. Bach: Grand challenge on breast cancer histology images. *Med. Image Anal.* **56**, 122–139 (2019). 31226662 10.1016/j.media.2019.05.010

[48] Spanhol, F. A., Oliveira, L. S., Petitjean, C. & Heutte, L. A dataset for breast cancer histopathological image classification. *IEEE Trans. Biomed. Eng.* **63**, 1455–1462 (2015). 26540668 10.1109/TBME.2015.2496264

[49] Vahadane, A. et al. Structure-preserving color normalization and sparse stain separation for histological images. *IEEE Trans. Med. imaging* **35**, 1962–1971 (2016). 27164577 10.1109/TMI.2016.2529665

[50] Howard, A. et al. Searching for mobilenetv3. In *Proceedings of the IEEE/CVF international conference on computer vision*, 1314–1324 (IEEE, 2019).

[51] Dosovitskiy, A. et al. An image is worth 16x16 words: Transformers for image recognition at scale. *In International Conference on Learning Representations* (2021).

[52] Kang, M., Song, H., Park, S., Yoo, D. & Pereira, S. Benchmarking self-supervised learning on diverse pathology datasets. In *Proceedings of the IEEE/CVF Conference on Computer Vision and Pattern Recognition*, 3344–3354 (IEEE, 2023).

[53] Riasatian, A. et al. Fine-tuning and training of densenet for histopathology image representation using tcga diagnostic slides. *Med. Image Anal.* **70**, 102032 (2021). 33773296 10.1016/j.media.2021.102032

[54] Filiot, A. et al. Scaling self-supervised learning for histopathology with masked image modeling. *medRxiv* https://www.medrxiv.org/content/10.1101/2023.07.21.23292757v1 (2023).

[55] Sui, W. et al. Micropapillary bladder cancer: Insights from the national cancer database. *Bladder Cancer* **2**, 415–423 (2016). 28035322 10.3233/BLC-160066 PMC5181670

[56] Selvaraju, R. R. et al. Grad-cam: Visual explanations from deep networks via gradient-based localization. In *Proceedings of the IEEE international conference on computer vision*, 618–626 (IEEE, 2017).

[57] DeLair, D. et al. Morphologic spectrum of immunohistochemically characterized clear cell carcinoma of the ovary: a study of 155 cases. *Am. J. Surgical Pathol.* **35**, 36–44 (2011). 10.1097/PAS.0b013e3181ff400e 21164285

[58] Sangoi, A. R. et al. Interobserver reproducibility in the diagnosis of invasive micropapillary carcinoma of the urinary tract among urologic pathologists. *Am. J. Surgical Pathol.* **34**, 1367–1376 (2010). 10.1097/PAS.0b013e3181ec86b3 20717002

[59] Goodfellow, I. et al. Generative adversarial nets. In: *Advances in neural information processing systems*, 27 (NIPS, 2014).

[60] Kingma, D. P. & Ba, J. Adam: A method for stochastic optimization. *In International Conference on Learning Representations* (2015).

[61] He, K., Zhang, X., Ren, S. & Sun, J. Deep residual learning for image recognition. In *Proceedings of the IEEE conference on computer vision and pattern recognition*, 770–778 (IEEE, 2016).

