# Supplementary material for: Learning generalizable AI models for multi-center histopathology image classification
Source: NPJ Precis Oncol. 2024 Jul 19;8:151. doi: 10.1038/s41698-024-00652-4 (PMC11271637; doi:10.1038/s41698-024-00652-4)
Supplement: Supplementary file 1 — Supplementary File [file 41698_2024_652_MOESM1_ESM.pdf]

Supplementary Figure 1: p-value comparison. The p-value matrix compares the balanced accuracy of different methods using the Wilcoxon signed-rank test, where each cell reflects the p-value between the models in the row and column for **a)** the Ovarian dataset, **a)** the Pleural dataset, **a)** the Bladder dataset, and **a)** the Breast dataset.

Supplementary Figure 2: Representative patches. **a)** Source and **b)** target domains for the Ovarian **(first row)**, Pleural **(second row)**, and Bladder **(third row)** cancer datasets.

Supplementary Figure 3: FFT-Enhancer. **a)** An example of the importance of phase spectrum. The morphology and color of each combined image resemble its phase and amplitude spectrum images, respectively. **b)** Some examples of applying the FFT-Enhancer of images with different color spaces.

Supplementary Figure 4: k in VLAD. Balanced accuracy of the model across different values of k for VLAD encoding for Ovarian, Pleural, Bladder, and Breast datasets.

Supplementary Table 1: Slide-level classification performance comparison for Ovarian dataset

Supplementary Table 2: Slide-level classification performance comparison for Pleural dataset

Supplementary Table 3: Slide-level classification performance comparison for Bladder dataset

Supplementary Table 4: Slide-level classification performance comparison for Breast dataset

Supplementary Table 5: Slide-level classification performance comparison (balanced accuracy) for different backbones (MobileNet (Mob) and Vision Transformer (ViT)

Supplementary Table 6: Slide-level classification performance comparison (balanced accuracy) for experiments with CTransPath

Supplementary Table 7: Comparative Analysis of Training Times and Computational Resources where "Training Time" refers to the duration required for training per epoch, measured in minutes, and"Param." denotes the total parameter count, quantified in millions

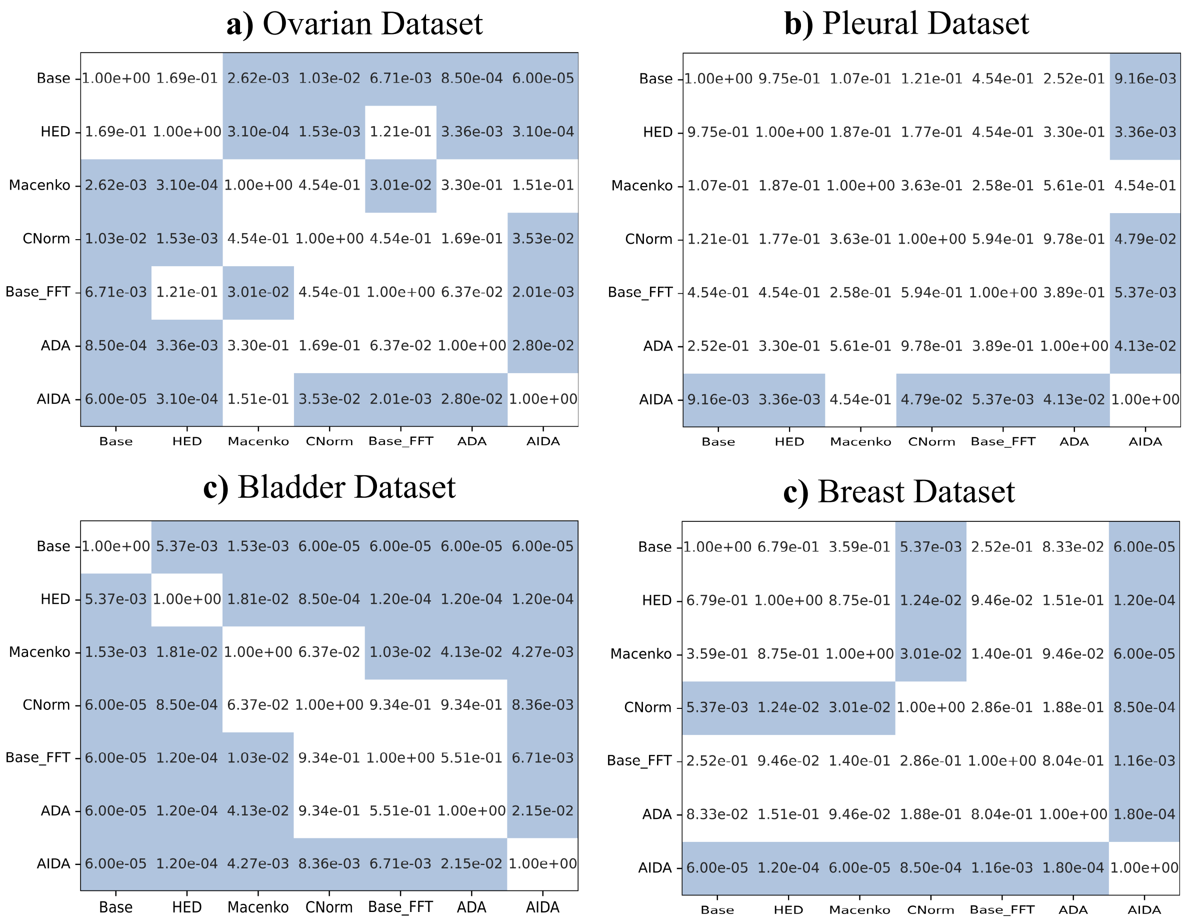

Supplementary Figure 1: p-value comparison. The p-value matrix compares the balanced accuracy of different methods using the Wilcoxon signed-rank test, where each cell reflects the p-value between the models in the row and column for **a)** the Ovarian dataset, **a)** the Pleural dataset, **a)** the Bladder dataset, and **a)** the Breast dataset.

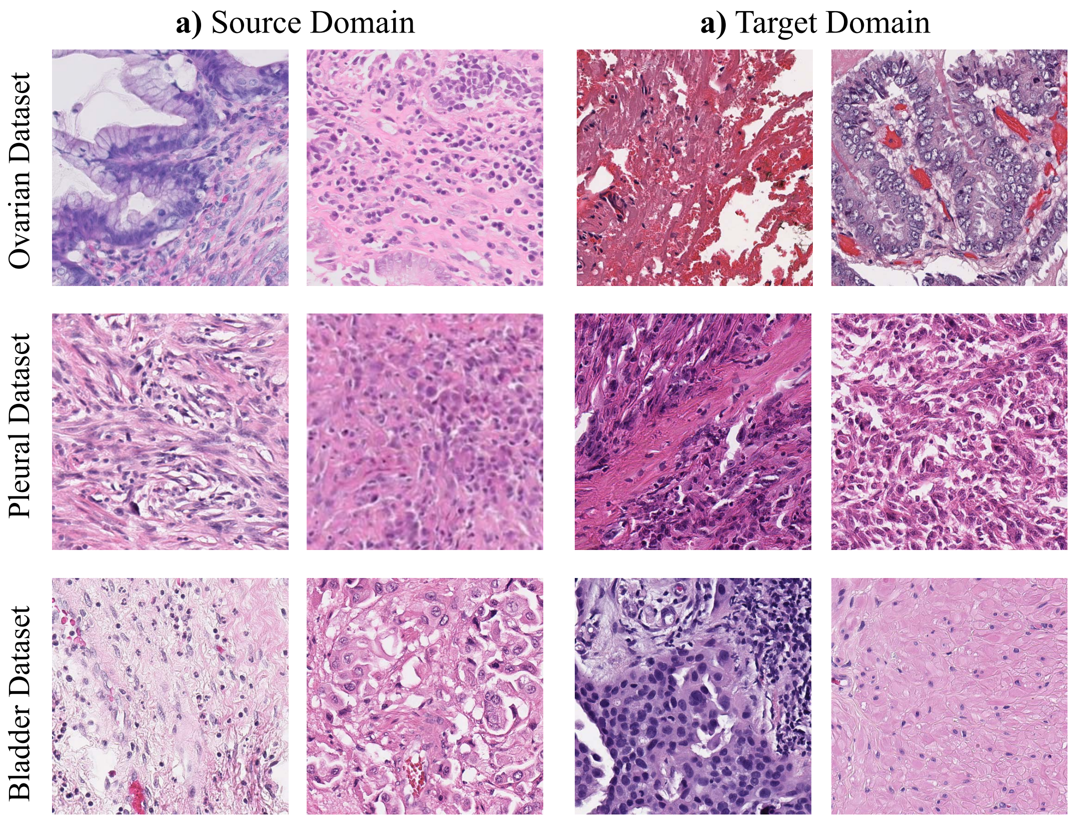

Supplementary Figure 2: Representative patches. **a)** Source and **b)** target domains for the Ovarian **(first row)**, Pleural **(second row)**, and Bladder **(third row)** cancer datasets.

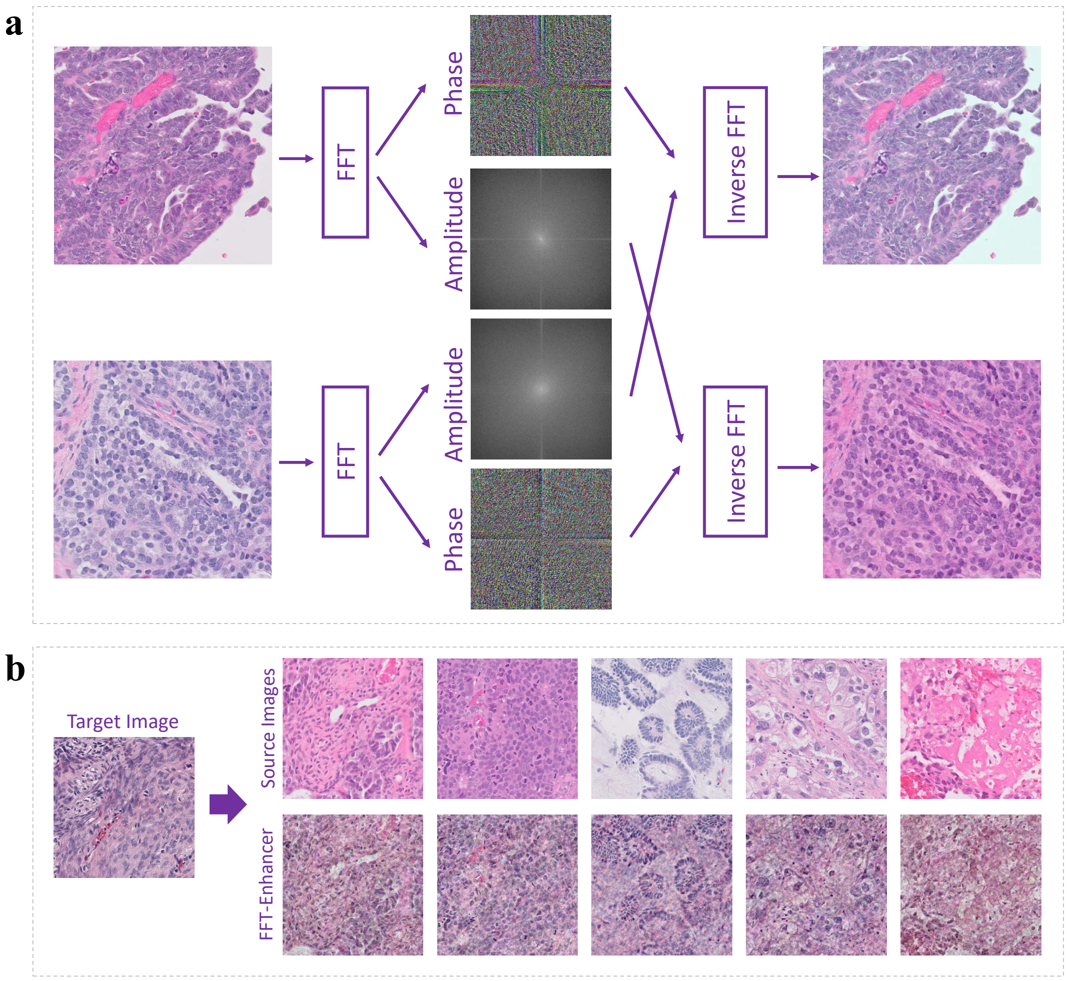

Supplementary Figure 3: FFT-Enhancer. **a)** An example of the importance of phase spectrum. The morphology and color of each combined image resemble its phase and amplitude spectrum images, respectively. **b)** Some examples of applying the FFT-Enhancer of images with different color spaces.

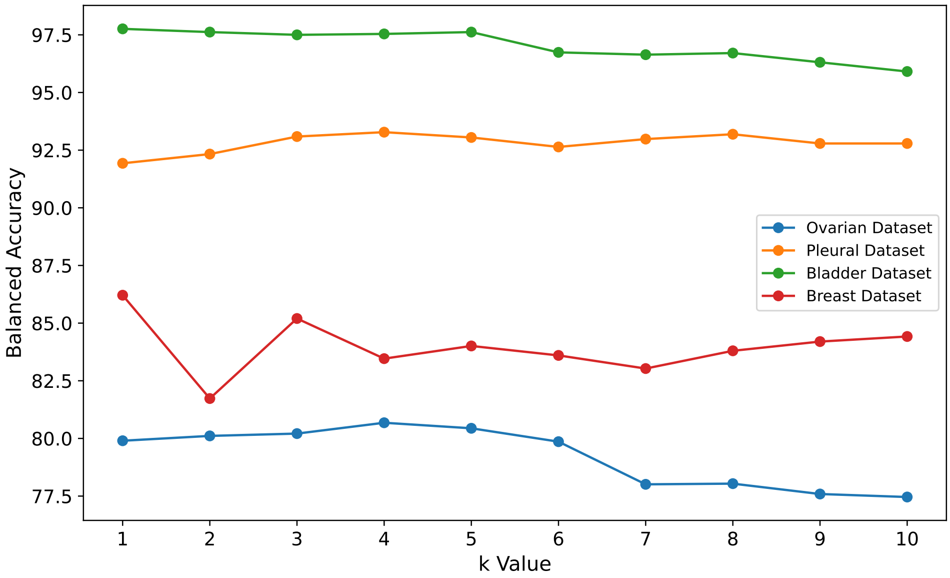

Supplementary Figure 4: k in VLAD. Balanced accuracy of the model across different values of k for VLAD encoding for Ovarian, Pleural, Bladder, and Breast datasets.

Supplementary Table 1: Slide-level classification performance comparison for Ovarian dataset

Split	Method	Source				Target			
		BACC	Kappa	F1	AUC	BACC	Kappa	F1	AUC
Split 1	Baseline	79.77	0.7246	0.7838	**0.9350**	61.08	0.6127	0.6131	0.8910
	HED[21]	79.07	0.7282	0.7814	0.9322	65.61	0.6583	0.6746	0.9056
	Macenko [26]	79.30	0.7102	0.7719	0.9319	73.56	0.6790	0.7400	0.9329
	CNorm [12]	80.62	0.7251	**0.7892**	0.9316	74.79	0.7165	0.7467	0.9300
	Baseline-FFT	80.71	**0.7343**	0.7826	0.9318	72.21	0.7026	0.7304	0.9284
	ADA	79.89	0.7250	0.7811	0.9297	75.49	0.7311	0.7525	0.9392
	**AIDA**	**81.10**	0.7313	0.7889	0.9329	**76.54**	**0.7588**	**0.7885**	**0.9429**
Split 2	Baseline	78.46	0.7060	0.7870	0.9445	71.29	0.6753	0.7191	0.9144
	HED[21]	78.77	**0.7162**	0.7960	0.9426	71.21	0.6940	0.7466	0.9378
	Macenko [26]	77.37	0.7010	0.7874	0.9429	74.13	0.6838	0.7543	**0.9442**
	CNorm [12]	76.86	0.6912	0.7726	0.9361	72.33	0.6764	0.7341	0.9327
	Baseline-FFT	77.60	0.7100	0.7851	0.9423	74.81	0.7194	0.7736	0.9408
	ADA	78.74	0.7133	0.7719	0.9318	77.95	0.7718	0.7999	0.9374
	**AIDA**	**78.92**	0.7158	**0.7994**	**0.9494**	**80.08**	**0.7917**	**0.8313**	0.9412
Split 3	Baseline	79.81	0.7055	0.7714	0.9483	61.59	0.5939	0.5986	0.8848
	HED[21]	79.63	0.7105	0.7835	0.9561	64.39	0.6357	0.6635	0.9168
	Macenko [26]	79.98	0.7162	0.7718	0.9518	**72.78**	0.6556	0.7189	0.9473
	CNorm [12]	79.94	0.7293	0.7866	0.9301	69.31	0.6464	0.6893	0.9344
	Baseline-FFT	78.74	0.7006	0.7732	0.9516	63.82	0.6448	0.6611	0.9288
	ADA	80.29	0.7329	0.7964	0.9536	69.03	0.6812	0.6968	0.9484
	**AIDA**	**82.02**	**0.7403**	**0.8073**	**0.9589**	70.84	**0.7053**	**0.7333**	**0.9492**
Average	Baseline	79.35	0.7120	0.7807	0.9426	64.65	0.6273	0.6436	0.8967
	HED[21]	79.16	0.7183	0.7869	0.9436	67.07	0.6627	0.6949	0.9201
	Macenko [26]	78.89	0.7091	0.7770	0.9422	73.49	0.6728	0.7377	0.9415
	CNorm [12]	79.14	0.7152	0.7828	0.9326	72.14	0.6798	0.7234	0.9324
	Baseline-FFT	79.02	0.7150	0.7803	0.9426	70.28	0.6889	0.7217	0.9327
	ADA	79.64	0.7237	0.7831	0.9384	74.16	0.7280	0.7831	0.9384
	**AIDA**	**80.68**	**0.7291**	**0.7985**	**0.9471**	**75.82**	**0.7519**	**0.7844**	**0.9444**

Supplementary Table 2: Slide-level classification performance comparison for Pleural dataset

Split	Method	Source				Target			
		BACC	Kappa	F1	AUC	BACC	Kappa	F1	AUC
Split 1	Baseline	**97.78**	**0.9580**	**0.9790**	**1.000**	82.69	0.6576	0.8198	**0.9920**
	HED[21]	95.96	0.9164	0.9581	0.9973	84.76	0.6970	0.8465	0.9524
	Macenko [26]	96.10	0.9174	0.9586	0.9957	82.71	0.6581	0.8236	0.9815
	CNorm [12]	95.91	0.9082	0.9434	0.9920	82.78	0.6584	0.8253	0.9413
	Baseline-FFT	96.42	0.9272	0.9636	0.9931	82.31	0.6656	0.8189	0.9658
	ADA	97.14	0.9381	0.9690	0.9941	83.13	0.6659	0.8282	0.9655
	**AIDA**	97.59	0.9483	0.9741	0.9984	**86.97**	**0.7423**	**0.8696**	0.9789
Split 2	Baseline	89.25	0.8005	0.9000	0.9783	81.62	0.6355	0.8121	0.9071
	HED[21]	**93.08**	**0.8590**	**0.9295**	0.9705	79.87	0.5991	0.7947	0.8960
	Macenko [26]	88.83	0.7898	0.8947	0.9617	83.97	0.6816	0.8378	**0.9630**
	CNorm [12]	82.42	0.6726	0.8401	0.9372	83.16	0.6660	0.8172	0.9313
	Baseline-FFT	89.92	0.8111	0.9050	0.9689	78.96	0.6210	0.7845	0.9254
	ADA	88.42	0.7802	0.8898	0.9639	83.20	0.6665	0.8299	0.9296
	**AIDA**	91.08	0.8250	0.9123	**0.9806**	**84.88**	**0.6979**	**0.8483**	0.9342
Split 3	Baseline	89.92	0.8269	0.9131	0.9628	65.78	0.3196	0.6175	0.8749
	HED[21]	89.43	0.8059	0.9026	0.9640	65.84	0.3197	0.6092	0.7832
	Macenko [26]	**94.20**	0.8981	**0.9490**	**0.9948**	**76.20**	**0.5279**	**0.7504**	0.9020
	CNorm [12]	93.80	**0.8988**	0.9431	0.9897	72.71	0.4896	0.7072	0.8932
	Baseline-FFT	90.14	0.8173	0.9083	0.9800	70.46	0.4356	0.6829	0.9006
	ADA	91.34	0.8495	0.9244	0.9486	72.83	0.4598	0.7160	0.8558
	**AIDA**	91.17	0.8297	0.9146	0.9863	75.83	0.5206	0.7485	**0.9268**
Average	Baseline	92.32	0.8618	0.9307	0.9804	76.70	0.5376	0.7498	0.9247
	HED[21]	92.82	0.8604	0.9301	0.9773	76.82	0.5386	0.7501	0.8772
	Macenko [26]	93.04	**0.8684**	0.9341	**0.9841**	80.96	0.6225	0.8039	**0.9488**
	CNorm [12]	90.71	0.8265	0.9089	0.9730	79.55	0.6047	0.7832	0.9219
	Baseline-FFT	92.16	0.8519	0.9256	0.9807	77.24	0.5741	0.7621	0.9306
	ADA	92.30	0.8559	0.9277	0.9689	79.72	0.5974	0.7914	0.9170
	**AIDA**	**93.28**	0.8677	0.9337	**0.9884**	**82.56**	**0.6536**	**0.8221**	0.9466

Supplementary Table 3: Slide-level classification performance comparison for Bladder dataset

Split	Method	Source				Target			
		BACC	Kappa	F1	AUC	BACC	Kappa	F1	AUC
Split 1	Baseline	**99.73**	**0.9928**	**0.9964**	**1**	53.46	0.0751	0.4316	0.6991
	HED[21]	99.28	0.9856	0.9928	1	60.22	0.2106	0.5456	0.7430
	Macenko [26]	99.50	0.9927	0.9964	1	69.34	0.3969	0.6734	0.8877
	CNorm [12]	99.09	0.9852	0.9926	**1**	69.27	0.4028	0.6744	**0.9317**
	Baseline-FFT	98.56	0.9675	0.9838	0.9982	71.16	0.4438	0.7049	0.8847
	ADA	96.76	0.9157	0.9577	**1**	69.42	0.4022	0.6797	0.8411
	**AIDA**	98.38	0.9577	0.9788	**1**	**73.36**	**0.4802**	**0.7343**	0.8561
Split 2	Baseline	95.90	0.9140	0.9567	0.9946	52.92	0.0636	0.4148	0.8686
	HED[21]	96.15	0.9203	0.9599	0.9997	53.90	0.0849	0.4434	0.7982
	Macenko [26]	**99.17**	**0.9840**	**0.9920**	**0.9999**	60.02	0.2117	0.5395	0.8916
	CNorm [12]	97.15	0.9406	0.9703	0.9975	76.01	0.5396	0.7547	**0.9647**
	Baseline-FFT	93.04	0.8594	0.9295	0.9919	75.69	0.5267	0.7457	0.9453
	ADA	89.66	0.7854	0.8915	0.9802	72.12	0.4613	0.7064	0.9415
	**AIDA**	94.91	0.8965	0.9481	0.9951	**80.89**	**0.6320**	**0.8091**	0.9483
Split 3	Baseline	98.62	0.9721	0.986	**1**	57.94	0.1709	0.5197	0.8515
	HED[21]	97.02	0.9413	0.9706	0.9986	58.85	0.1898	0.5375	0.8687
	Macenko [26]	96.55	0.9315	0.9656	0.9995	69.91	0.4137	0.6826	0.9149
	CNorm [12]	97.27	0.9443	0.9721	**1**	75.92	0.539	0.7578	**0.9631**
	Baseline-FFT	98.64	0.9721	0.986	0.9991	76.59	0.5497	0.7664	0.9408
	ADA	98.57	0.972	0.986	**1**	77.90	0.5716	0.7756	0.9372
	**AIDA**	**100**	**1**	**1**	**1**	**78.69**	**0.5886**	**0.7884**	0.9366
Average	Baseline	98.08	0.9596	0.9797	0.9982	54.77	0.1032	0.4554	0.8064
	HED[21]	97.48	0.9491	0.9744	0.9994	57.66	0.1618	0.5088	0.8033
	Macenko [26]	**98.41**	**0.9694**	**0.9847**	**0.9998**	66.42	0.3408	0.6318	0.8981
	CNorm [12]	97.84	0.9567	0.9783	**0.9992**	73.73	0.4938	0.7290	**0.9532**
	Baseline-FFT	96.75	0.9330	0.9664	0.9964	74.48	0.5067	0.7390	0.9236
	ADA	95.00	0.8910	0.9451	0.9934	73.15	0.4784	0.7206	0.9066
	**AIDA**	97.76	0.9514	0.9756	0.9984	**77.65**	**0.5669**	**0.7733**	0.9137

Supplementary Table 4: Slide-level classification performance comparison for Breast dataset

Split	Method	Source				Target			
		BACC	Kappa	F1	AUC	BACC	Kappa	F1	AUC
Split 1	Baseline	79.37	0.4248	0.6877	0.8302	53.17	0.0671	0.5129	0.4938
	HED[21]	**84.43**	**0.5279**	**0.7485**	0.8949	54.32	0.1093	0.5208	0.4648
	Macenko [26]	69.44	0.2479	0.5383	0.8455	51.66	0.0463	0.4487	0.6420
	CNorm [12]	79.59	0.5071	0.7395	0.9025	61.75	0.2805	0.6187	0.7555
	Baseline-FFT	70.42	0.2524	0.5488	0.8742	52.75	0.0750	0.4715	0.7126
	ADA	79.45	0.4157	0.6749	0.9382	54.35	0.1120	0.5151	0.7082
	**AIDA**	82.48	0.4857	0.7214	**0.9435**	**74.45**	**0.5002**	**0.7475**	**0.7892**
Split 2	Baseline	75.85	0.4253	0.6948	0.7903	54.28	0.0549	0.4808	0.5398
	HED[21]	81.53	0.5189	0.7441	0.8956	68.79	0.3676	0.6813	0.7212
	Macenko [26]	83.37	0.5844	0.7862	0.9109	68.87	0.3953	0.6938	0.8029
	CNorm [12]	**97.06**	**0.9258**	**0.9626**	**0.9969**	66.58	0.3772	0.6729	0.8158
	Baseline-FFT	83.82	0.5739	0.7776	0.9428	73.95	0.5130	0.7534	0.8326
	ADA	82.73	0.5424	0.7579	0.9337	69.64	0.3981	0.6967	0.7542
	**AIDA**	84.75	0.5926	0.7880	0.9356	**75.27**	**0.4943**	**0.7423**	**0.8474**
Split 3	Baseline	88.03	0.7601	0.8800	0.9167	58.00	0.1691	0.5730	0.5965
	HED[21]	89.46	0.7890	0.8945	0.9637	49.60	-0.0108	0.4344	0.5058
	Macenko [26]	88.00	0.7576	0.8784	0.9589	56.19	0.1513	0.5317	0.6878
	CNorm [12]	90.29	0.7639	0.8809	0.9641	66.84	0.3262	0.6522	0.7760
	Baseline-FFT	89.83	0.7963	0.8982	**0.9738**	55.56	0.1422	0.5204	0.7481
	ADA	**92.06**	**0.8401**	**0.9200**	0.9526	57.48	0.1855	0.5677	0.6619
	**AIDA**	91.39	0.8258	0.9126	0.9481	**72.60**	**0.4865**	**0.7411**	**0.8042**
Average	Baseline	81.08	0.5367	0.7542	0.8457	55.15	0.0971	0.5222	0.5434
	HED[21]	85.14	0.6119	0.7957	0.9181	57.57	0.1554	0.5455	0.5639
	Macenko [26]	80.27	0.5300	0.7343	0.9051	58.91	0.1976	0.5581	0.7109
	CNorm [12]	**88.98**	**0.7323**	**0.8610**	**0.9545**	65.06	0.3280	0.6479	0.7824
	Baseline-FFT	81.36	0.5408	0.7415	0.9303	60.75	0.2434	0.5818	0.7645
	ADA	84.75	0.5994	0.7843	0.9415	60.49	0.2319	0.5932	0.7081
	**AIDA**	86.21	0.6347	0.8073	0.9424	**74.11**	**0.4936**	**0.7436**	**0.8136**

Supplementary Table 5: Slide-level classification performance comparison (balanced accuracy) for different backbones (MobileNet (Mob) and Vision Transformer (ViT)

Split	Method	Ovarian		Pleural		Bladder		Breast	
		Source	Target	Source	Target	Source	Target	Source	Target
Split 1	ADA (Mob)	77.32	75.93	86.82	81.54	100	67.91	76.47	52.28
	**AIDA (Mob)**	**81.84**	**76.80**	**89.55**	**90.47**	**100**	**69.07**	**81.21**	**67.01**
	ADA (ViT)	80.5 0	71.19	93.96	89.30	**99.00**	74.48	78.87	56.91
	**AIDA (ViT)**	**81.30**	**73.10**	**94.55**	**90.11**	98.56	**76.21**	**79.84**	**61.90**
Split 2	ADA (Mob)	71.19	69.87	95.33	83.73	87.44	69.48	80.13	52.16
	**AIDA (Mob)**	**72.85**	**70.73**	**96.42**	**86.71**	**87.44**	**77.76**	**80.83**	**74.78**
	ADA (ViT)	76.18	72.99	95.42	86.71	93.08	68.52	79.58	71.18
	**AIDA (ViT)**	**77.19**	**72.66**	**96.67**	**88.96**	**94.06**	**69.86**	**80.49**	**73.04**
Split 3	ADA (Mob)	79.32	70.62	91.72	82.42	97.60	77.69	**90.26**	55.80
	**AIDA (Mob)**	**80.05**	**75.75**	**95.29**	**87.69**	**97.62**	**82.38**	88.07	**68.95**
	ADA (ViT)	79.68	64.64	92.83	79.76	**98.05**	80.06	89.87	56.18
	**AIDA (ViT)**	**82.27**	**70.87**	**96.09**	**85.01**	96.62	**81.47**	**90.58**	**61.35**
Average	ADA (Mob)	75.94	72.14	91.29	82.56	95.01	71.69	82.29	53.41
	**AIDA (Mob)**	**78.25**	**74.43**	**93.75**	**86.53**	**95.02**	**76.40**	**83.37**	**70.25**
	ADA (ViT)	78.79	69.61	94.07	85.26	**96.71**	74.35	82.77	61.42
	**AIDA (ViT)**	**80.25**	**72.21**	**95.77**	**88.03**	96.41	**75.85**	**83.64**	**65.43**

Supplementary Table 6: Slide-level classification performance comparison (balanced accuracy) for experiments with CTransPath

Split	Method	Ovarian		Pleural		Bladder		Breast	
		Source	Target	Source	Target	Source	Target	Source	Target
Split 1	CTransPath [46]	67.76	47.21	87.97	73.08	98.04	**74.32**	74.77	60.01
	ADA-CTransPath-Aug	80.61	79.06	94.79	75.09	**100**	59.96	81.91	74.11
	ADA-CTransPath-NoAug	77.39	70.09	93.05	78.45	98.26	59.83	82.97	57.32
	AIDA-CTransPath-Aug	81.12	79.41	94.01	76.37	**100**	60.66	81.49	71.26
	AIDA-CTransPath-NoAug	**81.40**	**83.77**	**95.70**	**78.69**	98.26	59.96	**82.96**	**76.21**
Split 2	CTransPath [46]	70.51	50.19	89.75	**77.38**	85.90	**86.15**	**80.60**	70.21
	ADA-CTransPath-Aug	82.31	81.82	94.75	66.47	90.51	65.72	80.26	80.80
	ADA-CTransPath-NoAug	77.90	77.19	96.42	60.78	85.64	66.74	78.28	79.07
	AIDA-CTransPath-Aug	**82.87**	**82.49**	**97.08**	69.22	**92.31**	60.66	78.26	**80.53**
	AIDA-CTransPath-NoAug	82.16	81.54	95.42	70.48	88.72	67.7	78.28	79.07
Split 3	CTransPath [46]	69.64	50.83	75.91	56.92	88.10	**79.14**	79.96	63.56
	ADA-CTransPath-Aug	79.00	80.13	93.86	68.37	98.07	62.71	89.80	62.31
	ADA-CTransPath-NoAug	70.13	73.71	**94.57**	66.90	90.95	65.25	85.42	74.53
	AIDA-CTransPath-Aug	**80.33**	80.90	93.54	**73.25**	95.69	64.66	**92.07**	73.91
	AIDA-CTransPath-NoAug	76.86	**82.71**	93.72	72.14	**98.10**	66.02	87.98	**77.07**
Average	CTransPath [46]	69.30	49.41	84.54	69.13	90.68	**79.87**	78.44	64.60
	ADA-CTransPath-Aug	80.64	80.34	94.47	69.98	**96.19**	62.71	**83.99**	72.41
	ADA-CTransPath-NoAug	75.14	73.66	94.68	68.71	91.62	65.25	81.22	67.74
	AIDA-CTransPath-Aug	**81.44**	80.93	94.88	72.95	96.00	63.42	83.94	75.23
	AIDA-CTransPath-NoAug	80.14	**82.67**	**94.95**	**73.77**	95.03	64.56	83.07	**77.45**

Supplementary Table 7: Comparative Analysis of Training Times and Computational Resources where “Training Time” refers to the duration required for training per epoch, measured in minutes, and“Param.” denotes the total parameter count, quantified in millions

**Model**	**Param. (millions)**	**FLOPs**	**Training Time**
Base	11	9.51×10^9	7
AIDA	78	9.58×10^9	10
